# Vildagliptin and Omarigliptin Differentially Bind to DPP‐4 Homodimers and Modulate Osteoclast‐Mediated Bone Resorption

**DOI:** 10.1002/cph4.70103

**Published:** 2026-01-21

**Authors:** Ratchaneevan Aeimlapa, Jiraporn Panmanee, Jarinthorn Teerapornpuntakit, Kannikar Wongdee, Jirawan Thongbunchoo, Nattapon Panupinthu, Saovaros Svasti, Nattayaporn Apaijai, Piangkwan Sa‐nguanmoo, Siriporn Chattipakorn, Nipon Chattipakorn, Narattaphol Charoenphandhu

**Affiliations:** ^1^ Center of Calcium and Bone Research (COCAB), Faculty of Science Mahidol University Bangkok Thailand; ^2^ Department of Physiology, Faculty of Science Mahidol University Bangkok Thailand; ^3^ Research Center for Neuroscience, Institute of Molecular Biosciences Mahidol University Nakhon Pathom Thailand; ^4^ Physiology Division, Preclinical Science, Faculty of Medicine Thammasat University Pathum Thani Thailand; ^5^ Faculty of Allied Health Sciences Burapha University Chonburi Thailand; ^6^ Thalassemia Research Center, Institute of Molecular Biosciences Mahidol University Nakhon Pathom Thailand; ^7^ Cardiac Electrophysiology Research and Training Center, Faculty of Medicine Chiang Mai University Chiang Mai Thailand; ^8^ Department of Physiology, Faculty of Medicine Chiang Mai University Chiang Mai Thailand; ^9^ Department of Physical Therapy, Faculty of Associated Medical Sciences Chiang Mai University Chiang Mai Thailand; ^10^ Department of Oral Biology and Diagnostic Sciences, Faculty of Dentistry Chiang Mai University Chiang Mai Thailand; ^11^ The Academy of Science The Royal Society of Thailand Bangkok Thailand; ^12^ Institute of Molecular Biosciences Mahidol University Nakhon Pathom Thailand

**Keywords:** bone histomorphometry, bone loss, diabetes mellitus, dipeptidyl peptidase‐4 (DPP‐4) inhibitor, holotomography, *in silico* molecular dynamics, osteoblast

## Abstract

Increased fracture risk in prediabetes and diabetes mellitus partly arises from bone collagen damage and enhanced bone resorption. Certain antidiabetic agents—particularly thiazolidinediones—paradoxically aggravate bone loss and fractures, especially in postmenopausal women with osteoporosis. However, dipeptidyl peptidase‐4 (DPP‐4) inhibitors (e.g., vildagliptin and omarigliptin) might help prevent diabetic osteopathy, although variable outcomes have been observed due to unknown mechanisms. Herein, we used high‐fat diet‐fed rats to demonstrate that oral administration of vildagliptin for 4 weeks not only alleviated insulin resistance but also improved tibial bone microstructure, as determined by bone histomorphometry. Further in vitro investigations in primary osteoblasts showed that both vildagliptin and omarigliptin similarly increased osteoblast viability, rather than upregulating the expression of osteoblast‐specific genes (e.g., Runx2 and alkaline phosphatase). We also used primary multinucleated osteoclasts to elucidate how the two DPP‐4 inhibitors modulated osteoclast functions. Interestingly, only omarigliptin, but not vildagliptin, reduced the number of TRAP‐positive cells and the mRNA expression of osteoclast‐specific genes (e.g., RANK and cathepsin K). *In silico* molecular dynamics revealed that omarigliptin and vildagliptin interacted differently with the DPP‐4 homodimer. Transient binding to one chain and tight binding to the other chain of the DPP‐4 homodimer by omarigliptin may be associated with its higher potency in inhibiting bone resorption. In conclusion, DPP‐4 inhibitors could improve bone microstructure, in part by increasing osteoblast viability and inhibiting osteoclast‐mediated bone resorption. Thus, omarigliptin may offer greater benefits to diabetic patients with osteoporosis, as it also helps suppress osteoclastogenesis and bone resorption.

## Graphical abstract

Vildagliptin and omarigliptin, dipeptidyl peptidase‐4 (DPP‐4) inhibitors, have differential effects on bone cells. Although vildagliptin improved the bone microstructure of high‐fat diet‐fed rats, it was unable to downregulate osteoclastogenesis or the expression of key osteoclast transcripts. Conversely, omarigliptin effectively attenuated bone resorption by suppressing osteoclast differentiation, maturation, and resorptive activity.

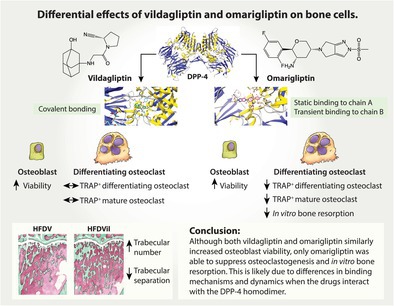

## 1 Introduction

Diabetes mellitus (DM) is a metabolic disorder with aberrant calcium and bone metabolism (Aeimlapa et al. 2018, 2019). In type 1 diabetes mellitus (T1DM), low bone mineral density (BMD) is commonly observed due to the absence or reduction of insulin signaling in bone. In contrast, BMD of patients with type 2 diabetes mellitus (T2DM) is varied, with studies reporting increases, decreases, or no change in BMD (Sosa et al. 1996; Tuominen et al. 1999; Kao et al. 2003; Al‐Maatouq et al. 2004; Strotmeyer et al. 2004). However, a high risk of bone fractures and poor bone quality was consistently evident in most T1DM and T2DM studies (Schwartz et al. 2001; Oei et al. 2013). Investigating the underlying mechanisms of bone defects in T2DM is challenging due to the multifactorial changes that occur during the progression of the disease. Moreover, our previous investigation in T2DM rats has suggested that glycemic control alone has not been sufficient to mitigate T2DM‐associated bone defects (Aeimlapa et al. 2018). Although most anti‐diabetic agents can lower blood glucose levels and prevent diabetic complications in several organs (e.g., heart, brain, kidney), some may either aggravate bone defects or mitigate bone loss in T2DM.

Among the various anti‐diabetic agents, dipeptidyl peptidase‐4 (DPP‐4) inhibitors have been reported to provide beneficial effects on bone health (Monami et al. 2011; Eom et al. 2016). They have only trivial effects on fasting plasma glucose in the prediabetic state (Rosenstock et al. 2008; Tanajak et al. 2017; Charoenphandhu et al. 2018), and pose a low risk of hypoglycemia by maintaining appropriate activities of insulin and glucagon (Farngren et al. 2012). DPP‐4 protein, also known as CD26, is an enzyme that generally terminates the action of incretins—a group of glucose‐lowering gastrointestinal hormones, namely glucose‐dependent insulinotropic polypeptide (GIP) and glucagon‐like peptide (GLP)‐1 (for a review, please see Drucker 2006)—as well as some other bioactive peptides such as neuropeptide Y, vasoactive intestinal peptide (VIP), substance P, and insulin‐like growth factor (IGF)‐1. Therefore, DPP‐4 inhibitors cause a prolonged elevation of GIP and GLP‐1 levels, which subsequently reduces blood glucose levels through incretin‐mediated insulin secretion in response to ingested nutrients, particularly glucose.

The positive effects of DPP‐4 inhibitors on bone health are not fully understood and may result from several mechanisms. For instance, the elevation of GIP and GLP‐1 proteins may indirectly stimulate bone formation and inhibit bone resorption by acting on GIP and GLP‐1 receptors on bone cells (Zhong et al. 2007; Mieczkowska et al. 2015). Additionally, the increase in circulating insulin induced by DPP‐4 inhibitor treatment might enhance osteoblast proliferation and differentiation (Yang et al. 2010). In parallel, osteoclasts are likely to be a direct target of DPP‐4 inhibitors. A previous study demonstrated that DPP‐4/CD26 is expressed by both osteoclast precursors and mature osteoclasts, and that its expression progressively increases during osteoclast differentiation (Nishida et al. 2014). A human monoclonal antibody against CD26 was shown to suppress osteoclastogenesis, osteoclast‐specific gene expression [e.g., tartrate‐resistant acid phosphatase (TRAP) and cathepsin K], and osteoclast activity (Nishida et al. 2014). Furthermore, Lee et al. (2025) demonstrated the expression and functional roles of osteoclast‐derived DPP‐4 in the regulation of osteoclast differentiation, which are conserved in both murine and human systems.

Although DPP‐4 is actually expressed in both osteoblasts and osteoclasts (Stanley et al. 2006; Nishida et al. 2014), it remains unclear whether DPP‐4 inhibitors—such as vildagliptin and omarigliptin—directly act on bone cells to modulate their functions. The observation that DPP‐4 inhibitors can restore bone turnover markers suggests potential effects on bone cells (Eom et al. 2016; Ishida et al. 2019). DPP‐4 inhibitors also have diverse chemical structures and pharmacodynamics (for a review, please see (Neumiller 2009; Nabeno et al. 2013)), which probably affect the responses of osteoblasts and osteoclasts. Therefore, the present study aimed to investigate the effects of the DPP‐4 inhibitor vildagliptin on bone microstructure in high‐fat diet (HFD)‐fed rats, which were used as a prediabetic animal model. Previously, HFD was able to decrease osteoblast‐related parameters and increase osteoclast parameters, leading to low bone mass (Eaimworawuthikul et al. 2019). It was evident that HFD was associated with low levels of bone formation markers (e.g., P1NP, alkaline phosphatase, osteocalcin), and high bone resorption markers (e.g., TRAP and CTX) (Chen et al. 2019). Furthermore, we also examined the direct effects of vildagliptin and omarigliptin on primary osteoblasts and osteoclasts isolated from mouse long bones. This study may provide insights into the underlying changes in bone cells in response to DPP‐4 inhibitors.

## 2 Materials and Methods

### 2.1 Animals

Adult male Wistar rats (7 weeks old, weighing 180–200 g) were obtained from the National Animal Center, Salaya Campus, Mahidol University. All rats were housed in a strictly controlled environment with a temperature of ~25°C and humidity of 50%–60%, under a 12:12‐h light–dark cycle. Before the experiment, they were fed a standard laboratory chow (Mouse Feed Food No. 082; C.P. Company, Bangkok, Thailand) containing ~20% energy from fat, providing a total energy of 4.02 kcal/g, and reverse‐osmosis (RO) water *ad libitum*. Body weights and food intake were recorded daily. After a 1‐week acclimatization period, the rats in the HFD‐fed group were switched from a standard laboratory chow to a HFD containing 59.28% energy from fat, including casein (250 g/kg), lard (310 g/kg), cholesterol (10 g/kg), vitamins (60 g/kg), dl‐methionine (3 g/kg), yeast powder (1 g/kg), and sodium chloride (1 g/kg), providing a total energy of 5.35 kcal/g.

Regarding the primary cell culture experiments, since our protocols for osteoblast and osteoclast cultures have been optimized for mice, as previously reported in Charoenphandhu et al. (2019), we decided to use mouse cells and were able to confirm that mouse pre‐osteoclasts could differentiate into their final stage, that is, multinucleated osteoclasts with TRAP expression. Briefly, female C57BL/6 mice (8 weeks old) obtained from the Thalassemia Research Center, Institute of Molecular Biosciences, Mahidol University, were used for bone cell isolation. The mice were housed in polystyrene cages under a 12:12‐h light–dark cycle, at a room temperature of 21°C–23°C, and a humidity of 50%–60% in an Association for Assessment and Accreditation of Laboratory Animal Care International (AAALAC)‐accredited facility. They were fed standard laboratory chow containing 1.0% calcium and 0.9% phosphorus (C.P. Company, Thailand) and given RO water *ad libitum*.

After a 1‐week acclimatization period, 9‐week‐old animals were anesthetized by the slow intraperitoneal injection of xylazine (0.125 mg/animal; L.B.S. Laboratory, Bangkok, Thailand) and tiletamine/zolazepam (1 mg/animal; Virbac Laboratories, Carros, France), during which surgical tissue collections were performed. Animals were euthanized under xylazine (5 mg/kg i.p.) and tiletamine/zolazepam (40 mg/kg i.p.) followed by cardiac removal. All experimental protocols, including those for anesthesia and euthanasia, were approved by the Institutional Animal Care and Use Committee (IACUC) of the Faculty of Medicine, Chiang Mai University, and the Faculty of Science, Mahidol University. All studies were conducted in accordance with relevant guidelines and regulations, including the ARRIVE guidelines (Animal Research: Reporting of in vivo Experiments; https://arriveguidelines.org/) and the American Veterinary Medical Association (AVMA) Guidelines for the Euthanasia of Animals (2020 edition).

### 2.2 Experimental Design

**FIGURE 1:**
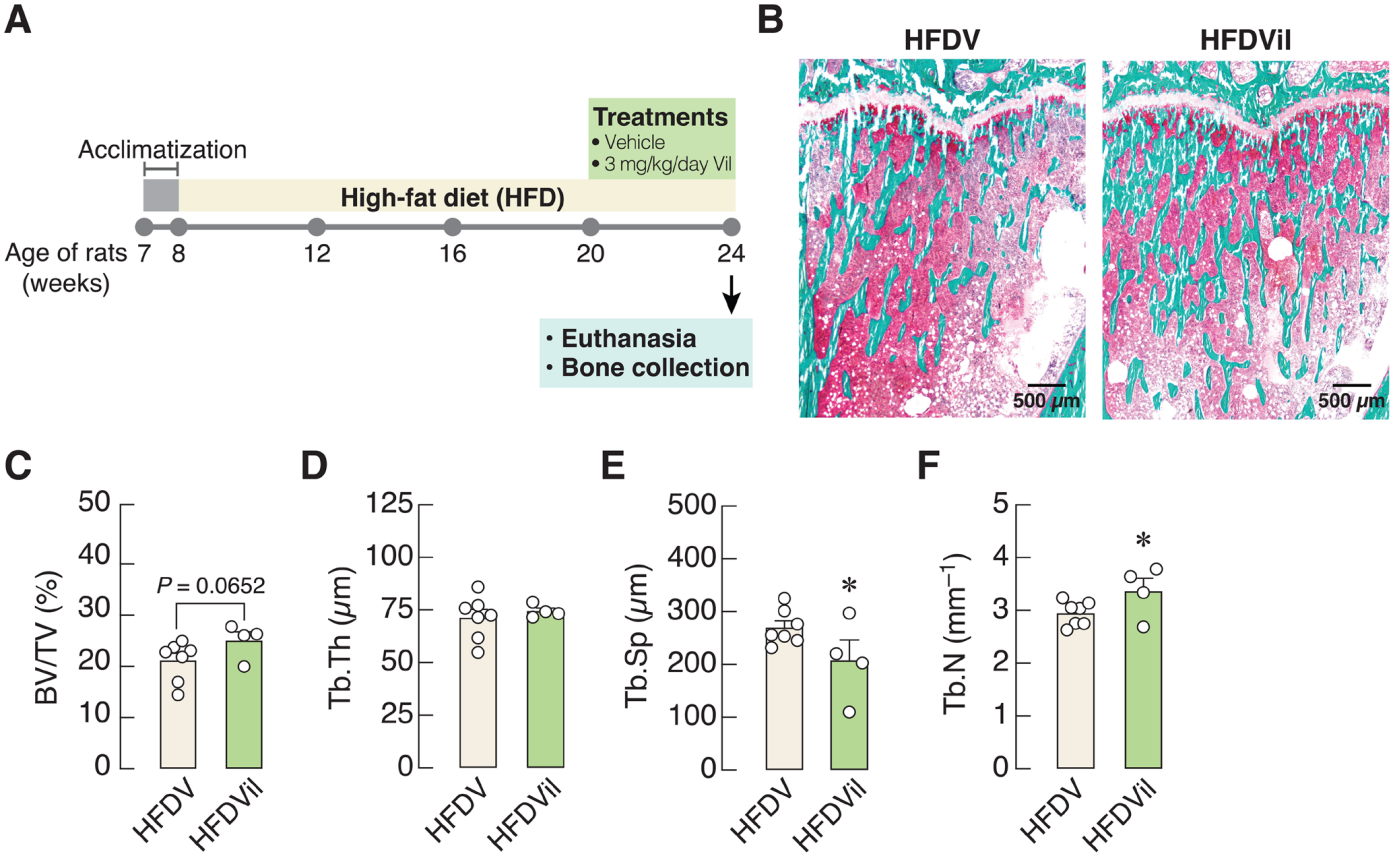
(A) A schematic picture shows anti‐diabetic drug treatment in high‐fat diet (HFD)‐induced prediabetic rats. (B) Representative photomicrographs of proximal tibial metaphysis of HFD fed rats after treated with vildagliptin (HFDVil) compared with vehicle treatment (HFDV). Resin‐embedded sections were stained with Goldner's trichrome. Microstructural analysis of proximal tibial metaphysis was determined by bone histomorphometry. (C) Trabecular bone volume fraction (BV/TV), (D) trabecular thickness (Tb.Th), (E) trabecular separation (Tb.Sp), (F) trabecular number (Tb.N) were assessed by computer‐assisted OsteoMeasure System. *n* = 4–7; *p < 0.05 vs. vehicle‐treated high‐fat diet‐fed rats (HFDV) by unpaired Student's *t*‐test.

As shown in Figure 1, the rats were randomly divided into the vehicle‐treated (HFDV) and vildagliptin‐treated (HFDVil) groups. In the HFDVil group, rats were orally given 3 mg/kg/day of vildagliptin for 4 weeks. Body weight and food intake were routinely recorded. At 16 weeks of age, plasma insulin levels were measured using an ELISA kit (Linco Research, St. Charles, MO, USA), while plasma glucose and lipid profiles were determined by colorimetric assays (Biotech, Bangkok, Thailand) after 6 h of fasting. Visceral fat and tibiae were collected for further analyzes. The homeostasis model assessment index (HOMA index) used to determine insulin resistance was calculated using Equation (1).

(1) $$\text{HOMA index}=\left(\text{fasting plasma insulin}\;\left(\mathrm{\mu U}/\mathrm{mL}\right)\right)\times \left(\text{fasting plasma glucose}\;\left(\text{mmol}/L\right)\right)/22.5$$

To investigate the direct effects of DPP‐4 inhibitors on bone cells, primary osteoblasts and osteoclast precursors were isolated from the tibiae and femora of female C57BL/6 mice, according to the modified methods of Marino et al. (2014) and Chevalier et al. (2021). The confluent osteoblasts were sub‐cultured and seeded in either 96‐well or 6‐well plates. After treatment with DPP‐4 inhibitors for 5 days, the cells were collected for studies on cell viability and mRNA expression, respectively. Furthermore, to examine the specific actions of DPP‐4 inhibitors on osteoclasts, the cells were exposed to each DPP‐4 inhibitor during pre‐osteoclast differentiation or maturation. Quantitative analyzes of multinucleated mature osteoclasts, osteoclast‐specific mRNA expression levels, and in vitro osteoclast activity were then performed.

### 2.3 Bone Histomorphometry

Tibiae were dehydrated in graded ethanol solutions of 70%, 95%, and 100% (vol/vol) for 3 days each. The bone samples were then embedded in methyl methacrylate resin at 42°C for 48 h. The resin‐embedded bone specimens were longitudinally cut into 7‐μm‐thick sections using a microtome equipped with a tungsten carbide blade (model RM2255; Leica, Nussloch, Germany). The bone sections were stained with Goldner's trichrome and later visualized under a light microscope (model BX51TRF; Olympus, Tokyo, Japan). Static bone histomorphometric parameters, including bone volume fraction (BV/TV), trabecular thickness (Tb.Th), trabecular separation (Tb.Sp), and trabecular number (Tb.N), were analyzed in the secondary spongiosa using the computer‐assisted OsteoMeasure System version 4.1 (OsteoMetrics, Atlanta, GA, USA).

### 2.4 Primary Osteoblast Culture

Primary osteoblasts were prepared from the mouse tibiae and femora. The osteoblast‐containing bone pieces were cultured in Dulbecco's modified Eagle's medium (DMEM; Sigma, St. Louis, MO, USA) supplemented with 30% fetal bovine serum (FBS; PAA Laboratories, Pasching, Austria), 100 U/mL penicillin–streptomycin (Gibco, Grand Island, NY, USA), and 0.1 mg/mL ascorbate‐2‐phosphate (Sigma, St. Louis, MO, USA). The cells were maintained in 5% CO_2_ at 37°C until they reached confluency. Only the first and second passages of confluent cells were used in this study.

### 2.5 Primary Osteoclast Culture

Primary osteoclasts were derived from the bone marrow cells of long bones from 9‐week‐old female mice. The osteoclast precursors were maintained in α‐MEM supplemented with 10% FBS and 10 ng/mL macrophage colony‐stimulating factor (M‐CSF) (catalog no. 216‐MC‐025; R&D Systems, Minneapolis, MN, USA) for 3 days. From days 3 to 16, the cells were further supplemented with 10 ng/mL M‐CSF and 10 ng/mL receptor activator of nuclear factor‐κB ligand (RANKL; catalog no. 462‐TEC‐010; R&D Systems) to induce osteoclast differentiation. Multinucleated osteoclasts were observed on days 7–8.

### 2.6 Cell Viability Test

The confluent osteoblasts were subcultured and seeded in a 96‐well plate (5 × 10^4^ cells/well). After 72 h, the cells were incubated with new culture media containing 1, 10, or 100 μM vildagliptin (catalog no. 14705; Cayman, Ann Arbor, MI, USA) or omarigliptin (catalog no. S8565; Selleckchem, Houston, TX, USA) for 5 days. The cells were then treated with 20 μL 3‐(4,5‐dimethylthiazol‐2‐yl)‐2,5‐diphenyltetrazolium bromide (MTT) reagent (5 mg/mL; catalog no. M2128; Sigma) and incubated at 37°C. After 3.5 h, the culture media were removed, and the cells were incubated with 150 μL of MTT solvent containing 4 mmol/L HCl and 0.1% vol/vol Nonidet P‐40 in isopropanol. Absorbance was measured at 590 nm with a reference filter of 620 nm using a microplate reader (model 1420; Wallac, Turku, Finland). Cell viability was expressed as a percentage of the vehicle control by converting the absorbance of the vehicle wells to 100%.

### 2.7 Tartrate‐Resistant Acid Phosphatase (TRAP) Staining

The bone marrow cells were plated at 1 × 10^5^ cells/well in 96‐well plates and cultured in α‐MEM containing osteoclastogenic factors, that is, 10 ng/mL M‐CSF and 10 ng/mL RANKL. During the differentiation stage, the cells were treated with various concentrations of DPP‐4 inhibitors (1, 10, or 100 μM vildagliptin or omarigliptin) from days 3 to 8. During the mature stage, each DPP‐4 inhibitor was added from days 7 to 16. The cells were fixed with 10% neutral buffered formalin and incubated with TRAP chromogenic substrate (catalog no. PMC‐AK04F‐COS; Cosmo Bio, Tokyo, Japan) at 37°C for 60 min. TRAP‐positive multinucleated osteoclasts containing 3 or more nuclei were visualized and manually counted as previously described (Alatalo et al. 2000; Araujo et al. 2009).

### 2.8 Quantitative Real‐Time PCR

Primary osteoblasts and bone marrow cells were plated at 3 × 10^5^ and 5 × 10^5^ cells/well, respectively, in 6‐well plates. After 3 days of plating, the cells were treated with 1, 10, or 100 μM vildagliptin or omarigliptin for 5 days. On day 8, total RNA was extracted using TRIzol reagent (Invitrogen, Carlsbad, CA, USA). The concentration and purity of the total RNA were determined using a NanoDrop‐2000c spectrophotometer (Thermo Scientific, Waltham, MA, USA). An absorbance ratio between 1.8 and 2.0 at 260/280 nm was considered indicative of purified RNA. One microgram of total RNA was used as a template for cDNA synthesis with the iScript cDNA synthesis kit (catalog no. 170–8897; Bio‐Rad, Hercules, CA, USA). The primers used in this study were listed in Table S1. The specificity and efficiency of the primers were validated by conventional PCR and RNA sequencing. Reverse transcription and conventional PCR were performed using a thermal cycler (model MyCycler; Bio‐Rad). Quantitative real‐time PCR and melting curve analyzes were performed with SsoFast EvaGreen Supermix (Bio‐Rad) on a QuantStudio 3 Real‐Time PCR system (Applied Biosystems, Foster City, CA, USA) for 40 cycles at 95°C for 5 s and an annealing temperature of 54°C–60°C for 20 s. Mouse β‐actin and 18S rRNA were used as housekeeping genes for primary osteoblasts and osteoclasts, respectively. Changes in gene expression were calculated from the threshold cycles (C_t_).

### 2.9 Three‐Dimensional (3D) Holotomographic Imaging

Bone marrow cells were plated at 5 × 10^5^ cells/well in TomoDish (Tomocube Inc., Daejeon, South Korea) and cultured in α‐MEM containing osteoclastogenic factors (10 ng/mL M‐CSF and 10 ng/mL RANKL) for 7 days. The cells were then fixed with 4% paraformaldehyde at room temperature for 20 min. Following fixation, the nuclei were stained with 4′,6‐diamidino‐2‐phenylindole (DAPI). Cells were visualized and captured under a 60× objective lens with a numerical aperture of 1.2 (water immersion) using a holotomographic microscope equipped with a 3D fluorescence imaging system (model HT‐2H; Tomocube Inc.). The 3D hologram images were reconstructed based on the refractive index (RI) distribution of each cellular compartment using TomoStudio version 3.3.9 and TomoStitcher version 1.2.0 software (https://www.tomocube.com/products/ht2h). Cells with 3 or more nuclei were identified as primary osteoclasts.

### 2.10 Bone Resorption Pit Assay

Bone marrow cells were plated on bone slices (catalog no. DT‐1BON1000‐96; Immunodiagnostic Systems, Tyne and Wear, UK) at a density of 1 × 10^5^ cells/well in 96‐well plates and then cultured in α‐MEM containing osteoclastogenic factors for 16 days. On day 7, the multinucleated osteoclasts growing on the bone slices were incubated with 1 or 100 μM omarigliptin until day 16. The culture media were collected to determine the levels of C‐terminal telopeptides of type 1 collagen (CTX‐1) in the supernatant using a crosslaps for cell culture ELISA kit (catalog no. AC‐07F1; Immunodiagnostic Systems). The bone slices were sonicated in ammonium hydroxide for 4 min to remove the cells, then washed with distilled water. The bone slices were stained with 1% toluidine blue in 1% sodium borate for 10 min, washed with distilled water, and air‐dried. The resorption pits were captured using a light microscope (model BX51TRF; Olympus).

### 2.11 *In Silico* Molecular Docking and Molecular Dynamics Simulations of DPP‐4 Inhibitor Binding

The crystal structure of human DPP‐4 (PDB: 2QT9) was used as a template to generate the structure of the mouse DPP‐4 homodimer through homology modeling, utilizing the SWISS‐MODEL server (https://swissmodel.expasy.org). The binding energies (ΔG_binding_ in kcal/mol) of omarigliptin with mouse DPP‐4 (mDPP‐4), rat DPP‐4 (PDB: 2GBC), and human DPP‐4 (PDB: 4PNZ) were predicted using Autodock Vina. The protein structure was prepared for molecular docking using Dock Prep with default suite. The grid parameters were set as follows: center x, y, z = 41.79, 50.93, 36.89 Å, dimension x, y, z = 22.00, 22.00, 22.00 Å. The protein‐ligand complex was generated by using UCSF Chimera. The complex was then subjected to molecular dynamics (MD) simulations using Desmond version 6.9 (Schrödinger LLC) via Schrödinger Maestro suite version 13.1.137, utilizing an NVIDIA 80GB GPU at Mahidol University Artificial Intelligence Center (MUAI). The explicit TIP3P water model was used to solvate the system with 0.15 M NaCl, under periodic boundary conditions with a buffer distance of 10 Å. Additional Na^+^ and Cl^−^ ions were added to neutralize the total charge of the protein molecules. After a pre‐equilibration of 100 ps, the molecular dynamics simulation was run for 1 ms using the NPT ensemble and the OPLS_2005 force field. The temperature was maintained at 300 K using the Nosé‐Hoover chain method, and the pressure was maintained at 1.01 bar using the Martyna‐Tobias‐Klein method. Coulombic interactions were calculated with a cutoff radius of 9.0 Å. Trajectories were recorded at 2‐ns intervals. The protein‐ligand interactions were analyzed using the simulation interaction diagram and visualized with UCSF Chimera and the PyMOL Molecular Graphics System (Schrödinger LLC). The human DPP‐4 complexes with omarigliptin (PDB: 4PNZ) and vildagliptin (PDB: 3W2T) were retrieved and subjected to MD simulations, following the same protocol as that used for similar mouse DPP‐4.

### 2.12 Statistical Analysis

The results are presented as means ± standard error (SE). Two sets of data were compared using unpaired Student's *t*‐test. One‐way analysis of variance (ANOVA) with Dunnett's multiple comparisons test was used for multiple sets of independent data. The level of significance for statistical tests was *p* < 0.05. All data were analyzed by GraphPad Prism 9 (GraphPad Software, San Diego, CA, USA).

## 3 Results

### 3.1 Vildagliptin Improved Bone Microstructure of HFD‐Treated Prediabetic Rats

It has been known that feeding HFD for 12 weeks could induce prediabetes in rats with insulin resistance and hyperlipidemia (Tanajak et al. 2017; Apaijai et al. 2021; Maneechote et al. 2022). In the present study, vildagliptin treatment for 4 weeks effectively alleviated prediabetes and hyperlipidemia, as indicated by decreases in plasma insulin, HOMA index, plasma total cholesterol, and plasma low‐density lipoprotein levels (Table 1). As shown in Figure 1, Goldner's trichrome staining of bone specimens revealed a greater amount of mineralized tissue (green) in vildagliptin‐treated HFD rats, suggesting vildagliptin‐induced bone accretion. Bone histomorphometry (Figure 1) revealed that the vildagliptin‐treated HFD rats exhibited a decrease in trabecular separation (Tb.Sp) and an increase in trabecular number (Tb.N) without significant change in trabecular thickness (Tb.Th). Quantitative analysis of bone volume fraction (BV/TV) showed an increasing tendency in the HFDVil group compared to the HFDV group (25.02 ± 1.73 in HFDVil vs. 21.11 ± 1.47 in HFDV), but this difference did not reach statistical significance (*p* = 0.0652). Nevertheless, our findings could suggest that the DPP‐4 inhibitor vildagliptin had positive effects on bone microstructure.

**TABLE 1:** Effects of vildagliptin on metabolic parameters of high‐fat diet (HFD)‐fed rats for 4 weeks.

Parameters	HFDV	HFDVil
Body weight (g)	690 ± 13.0	676 ± 9.0
Visceral fat (g)	62 ± 1.0	62 ± 2.0
Food intake (g/day)	21 ± 1.0	19 ± 1.0
Urine glucose excretion (UGE; mg/dL)	1.3 ± 0.1	1.0 ± 0.2
Plasma insulin (ng/mL)	5.6 ± 0.2	3.9 ± 0.2^†^
Plasma glucose (mg/dL)	113 ± 4.0	115 ± 2.0
HOMA index	40.4 ± 2.7	27.5 ± 1.8^†^
AUCg (mg/dL × min × 10^4^)	4.0 ± 0.2	3.20 ± 0.1^†^
Plasma TC (mg/dL)	130 ± 6.0	105 ± 2.0^†^
Plasma TG (mg/dL)	88 ± 6.0	90 ± 7.0
Plasma HDL‐c (mg/dL)	33 ± 2.0	29 ± 2.0
Plasma LDL‐c (mg/dL)	84 ± 5.0	60 ± 4.0^†^

Abbreviations: HDL‐c, high‐density lipoprotein cholesterol; HFDV, high‐fat diet‐fed group treated with vehicle; HFDVil, high‐fat diet‐fed group treated with vildagliptin; LDL‐c, low‐density lipoprotein cholesterol; TC, total cholesterol; TG, triglyceride.

^†^ *p* < 0.05 vs. HFDV.

### 3.2 Vildagliptin and Omarigliptin Directly Increased Osteoblast Viability but Not Osteoblast Differentiation

**FIGURE 2:**
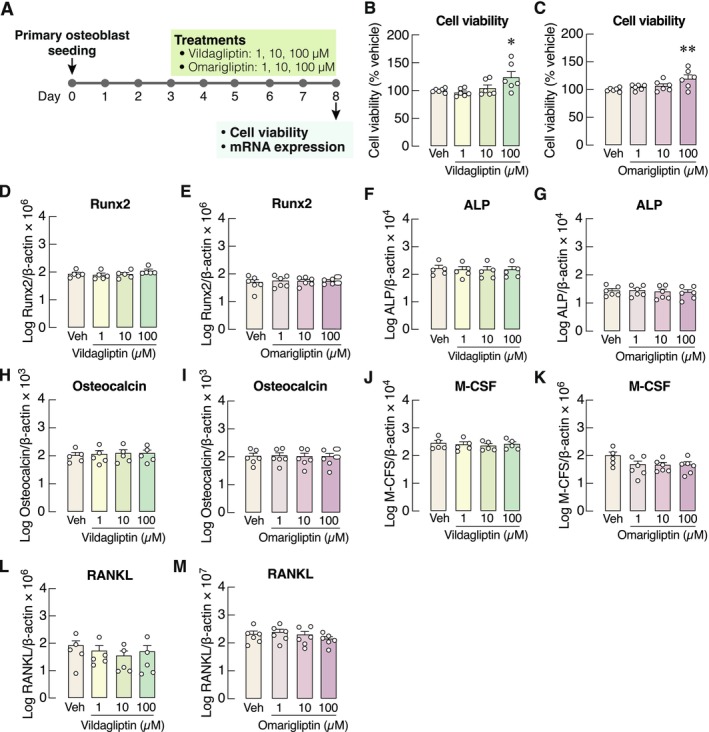
(A) A schematic picture shows the experimental plan for osteoblast viability and mRNA expression study. (B–C) Cell viability of primary osteoblasts after exposure to 1, 10, 100 μM vildagliptin or omarigliptin. mRNA expression of osteoblast‐specific genes, that is, (D–E) Runx2, (F–G) alkaline phosphatase (ALP), (H–I) osteocalcin, and osteoclastogenic factors, that is, (J–K) macrophage‐colony stimulating factor (M‐CSF), (L–M) receptor activator of nuclear factor‐κB ligand (RANKL) in primary osteoblasts after treatment with 1, 10, 100 μM vildagliptin or omarigliptin for 5 days. *n* = 5–6; *p < 0.05, **p < 0.01 vs. vehicle‐treated group (Veh) by one‐way analysis of variance (ANOVA) with Dunnett's multiple comparisons test.

To further elucidate whether DPP‐4 inhibitors induced bone gain through their direct effects on bone‐forming cells, we used primary mouse osteoblasts as a model for subsequent investigations (Figure 2). An in vitro study on primary osteoblasts found that cell viability increased by approximately 24% and 20% after treatment with 100 μM vildagliptin and 100 μM omarigliptin (Figure 2), respectively. However, both drugs had no direct effect on the mRNA expression levels of osteoblast differentiation markers, such as *runt*‐related transcription factor‐2 (Runx2), alkaline phosphatase (ALP), and osteocalcin. Similarly, the expression of osteoblast‐derived osteoclastogenic markers, that is, M‐CSF and RANKL (Figure 2), and cell cycle regulation markers, that is, cyclin D1, Cdkn1a and Cdkn2a (Figure S1), was not altered by vildagliptin or omarigliptin. Thus, it is likely that both DPP‐4 inhibitors help prolong the viability of osteoblasts rather than modulating the expression of osteoblast‐specific markers. Nevertheless, 1 μM vildagliptin modestly downregulated Bcl‐2 expression, whereas 10 and 100 μM vildagliptin and all doses of omarigliptin did not alter Bcl‐2 expression (Figure S1).

### 3.3 Omarigliptin, but Not Vildagliptin, Could Attenuate Osteoclast Differentiation and Function

**FIGURE 3:**
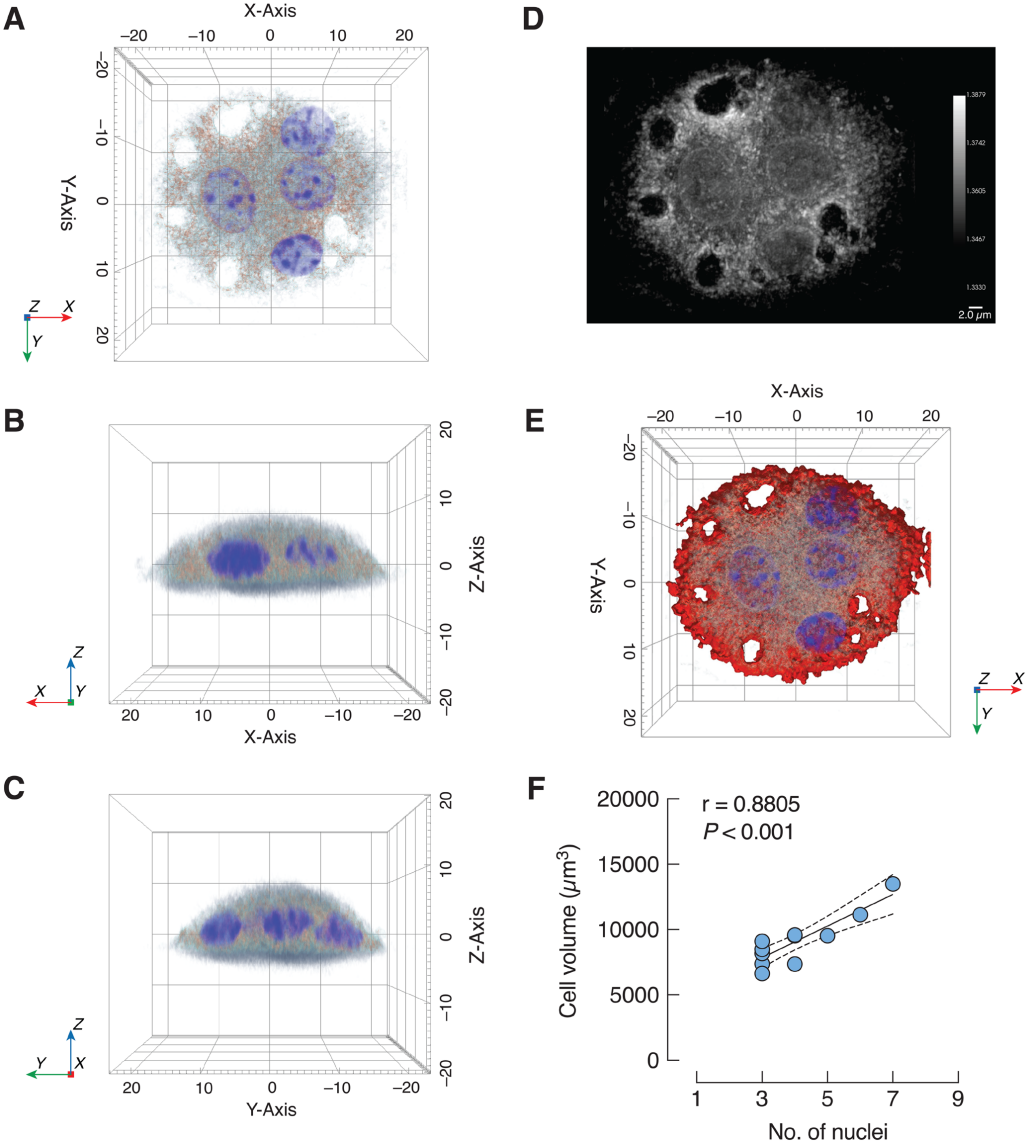
Representative images of primary osteoclast captured by holotomographic microscope. Osteoclast morphology on (A) x‐y, (B) x‐z, (C) y‐z axis at day 7 after culture bone marrow cells with 10 ng/mL M‐CSF and RANKL. (D) Refractive index (RI) distribution in primary osteoclasts, (E) three‐dimentional (3D) rendered image of RI distribution. Blue is DAPI stained nuclei. Red is pseudocolor labeling for the remaining of intracellular compartments. (F) Correlation between number of nuclei and cell volume of multinucleated osteoclasts.

**FIGURE 4:**
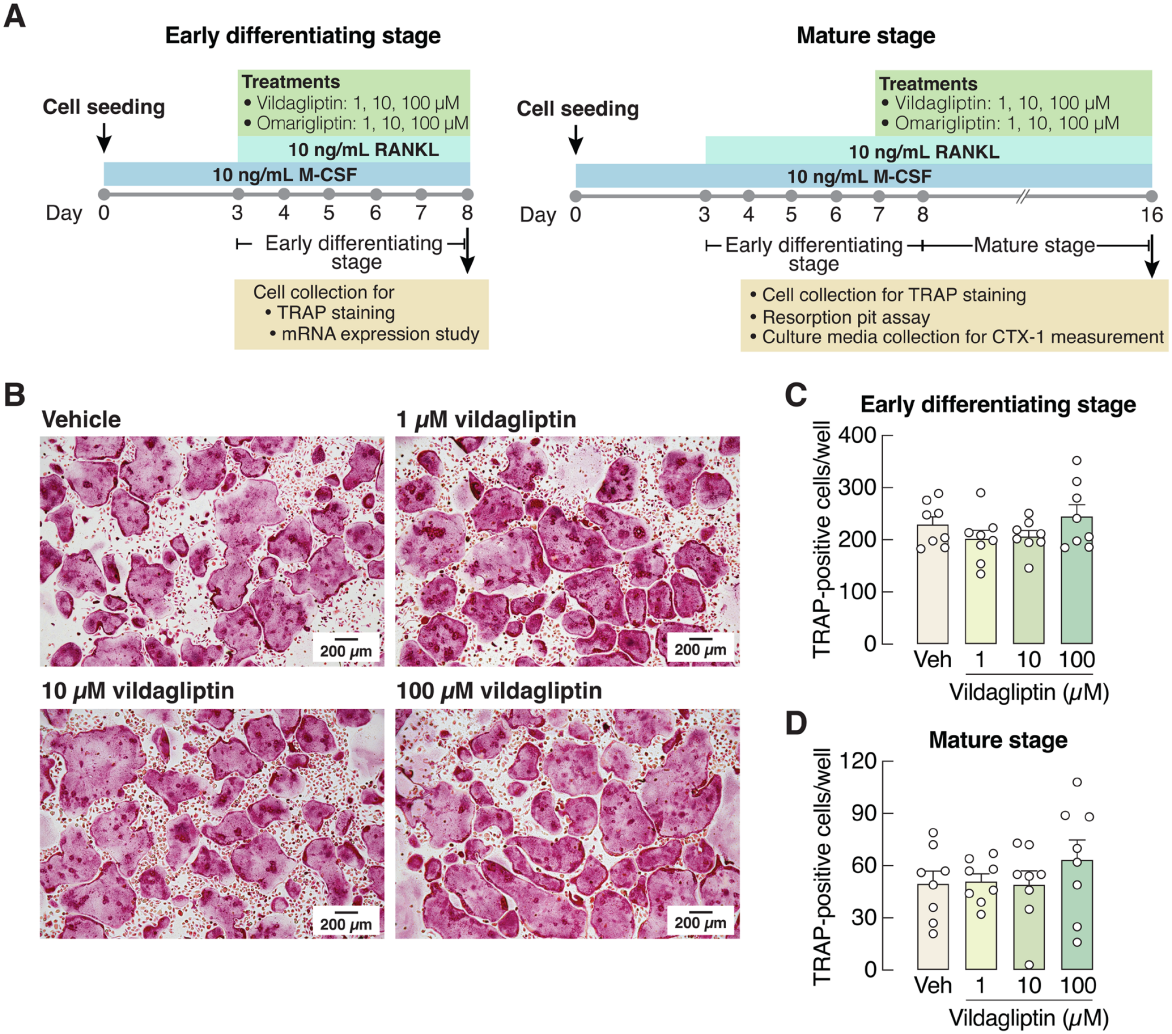
(A) A schematic picture shows experiment timeline for tartrate‐resistant acid phosphatase (TRAP) staining, mRNA expression study, resorption pit assay, and measurement of the released C‐terminal telopeptides of type 1 collagen (CTX‐1) in culture media. (B) Representative photomicrographs of TRAP‐positive multinucleated osteoclasts after culture for 8 days. (C–D) Number of TRAP‐positive cells was manually counted after 1, 10, 100 μM vildagliptin treatment during differentiating stage (day 3–8) or mature stage (day 7–16) of osteoclasts. *n* = 8.

In this series of experiments, primary osteoclasts were employed to determine whether DPP‐4 inhibitors could directly modulate osteoclast function. We successfully cultured primary osteoclasts derived from the bone marrow cells of mouse long bones. The three‐dimensional morphology of primary osteoclasts was visualized based on the refractive index of cellular components and captured using a holotomographic microscope (Figure 3). The surface area of mature osteoclasts measured 5950 ± 383.8 μm^2^ (*n* = 12 cells) with numerous finger‐like processes and multiple nuclei, the latter of which showed a positive correlation with cell volume (Figure 3). Therefore, the morphology of the primary cultured cells, along with TRAP staining, was consistent with differentiated mouse osteoclasts, ready for subsequent studies (Figure 4).

**FIGURE 5:**
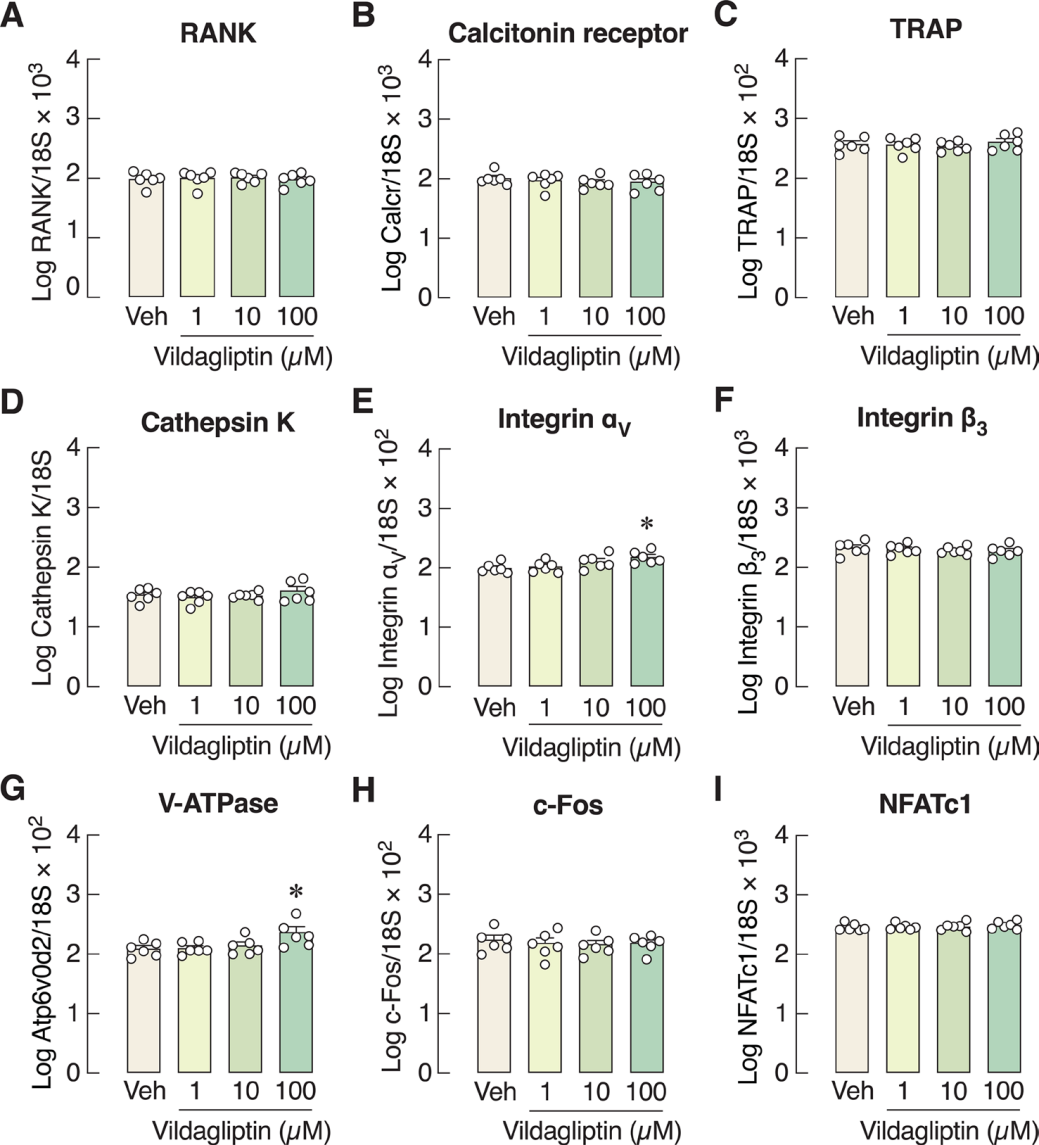
mRNA expression of osteoclast‐specific genes, that is, (A) receptor for receptor activator of nuclear factor‐κB ligand (RANK), (B) calcitonin receptor, (C) tartrate‐resistant acid phosphatase (TRAP), (D) cathepsin K, (E) integrin α_v_, (F) integrin β_3_, (G) vacuolar ATPase H^+^ Transporting V0 Subunit D2 (V‐ATPase), and signaling factors, that is, (H) c‐Fos, (I) nuclear factor of activated T‐cells c1 (NFATc1) after treatment with 1, 10, 100 μM vildagliptin for 5 days. *n* = 6; *p < 0.05 vs. vehicle‐treated group by one‐way analysis of variance (ANOVA) with Dunnett's multiple comparisons test.

Representative images and quantitative analysis revealed that the number of TRAP‐positive multinucleated osteoclasts was not altered by 1–100 μM vildagliptin during either early differentiating or mature stages (Figure 4). Consistently, the mRNA expression levels of most osteoclast‐specific markers and genes related to osteoclast functions were not affected by vildagliptin (Figure 5), except for integrin α_v_ and vacuolar H^+^ ATPase (H^+^‐transporting V0 subunit D2, also known as Atp6v0d2 or V‐ATPase), which were upregulated by 100 μM vildagliptin, but not by lower concentrations (Figure 5).

**FIGURE 6:**
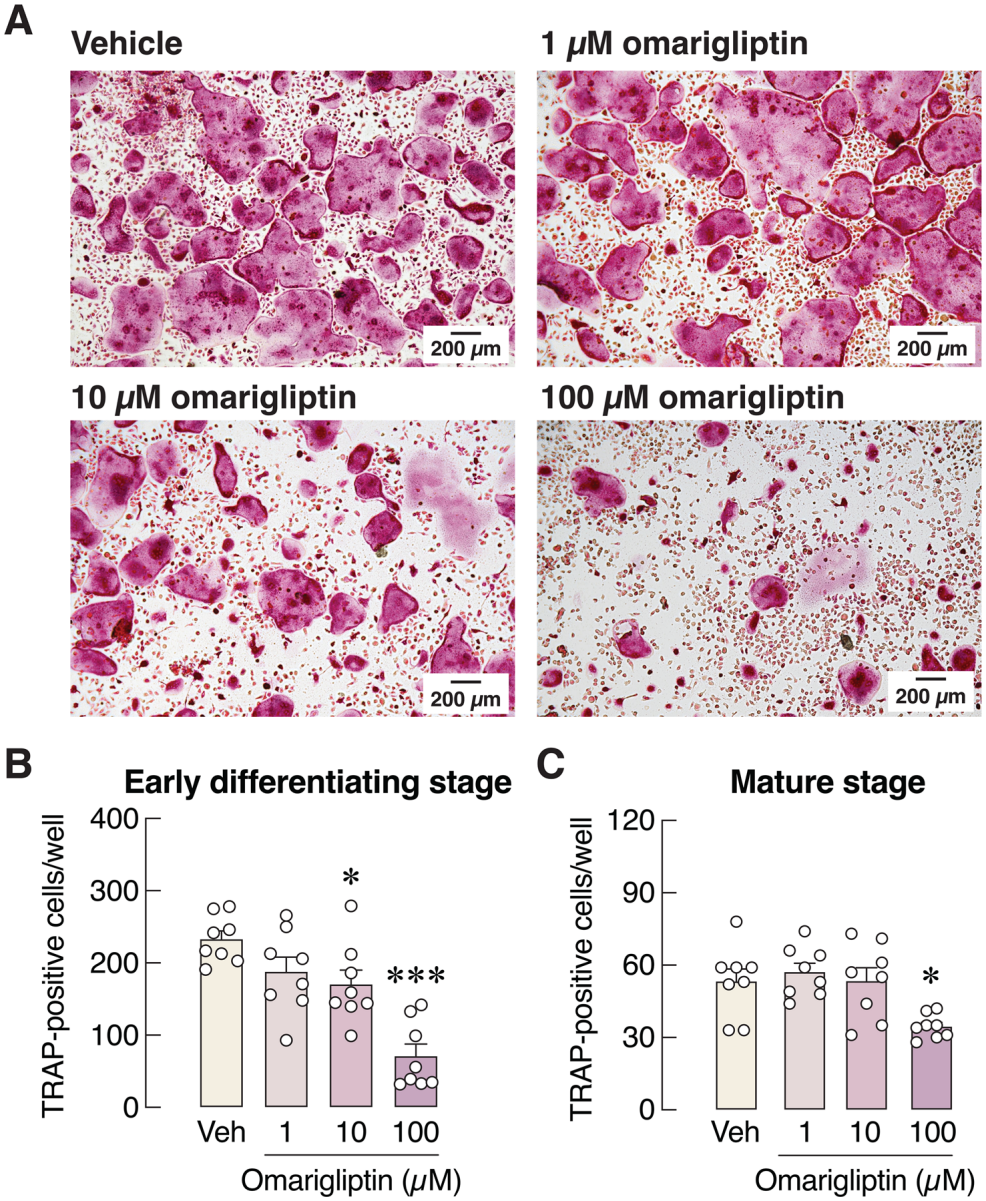
(A) Representative photomicrographs of TRAP‐positive multinucleated osteoclasts after culture for 8 days. Number of TRAP‐positive cells were manually counted after 1, 10, 100 μM omarigliptin treatment during (B) osteoclast differentiation (day 3–8) or (C) osteoclast maturation (day 7–16). *n* = 8; *p < 0.05, ***p < 0.001 vs. vehicle‐treated group by one‐way analysis of variance (ANOVA) with Dunnett's multiple comparisons test.

**FIGURE 7:**
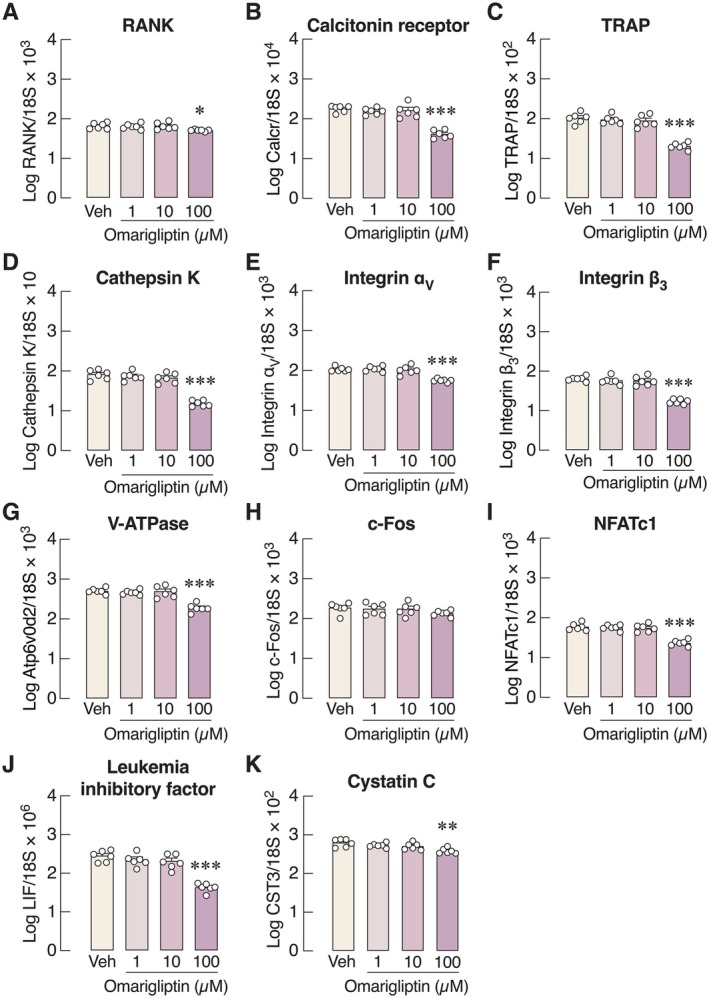
mRNA expression of osteoclast‐specific genes, that is, (A) receptor for receptor activator of nuclear factor‐κB ligand (RANK), (B) calcitonin receptor, (C) tartrate‐resistant acid phosphatase (TRAP), (D) cathepsin K, (E) integrin α_v_, (F) integrin β_3_, (G) vacuolar ATPase H^+^ Transporting V0 Subunit D2 (V‐ATPase), signaling factors, that is, (H) c‐Fos, (I) nuclear factor of activated T‐cells c1 (NFATc1), osteoclast‐derived coupling factors (J) leukemia inhibitory factor (LIF), and (K) cystatin C (CST3) after treatment with 1, 10, 100 μM omarigliptin for 5 days. *n* = 6; *p < 0.05, **p < 0.01, ***p < 0.001 vs. vehicle‐treated group by one‐way analysis of variance (ANOVA) with Dunnett's multiple comparisons test.

**FIGURE 8:**
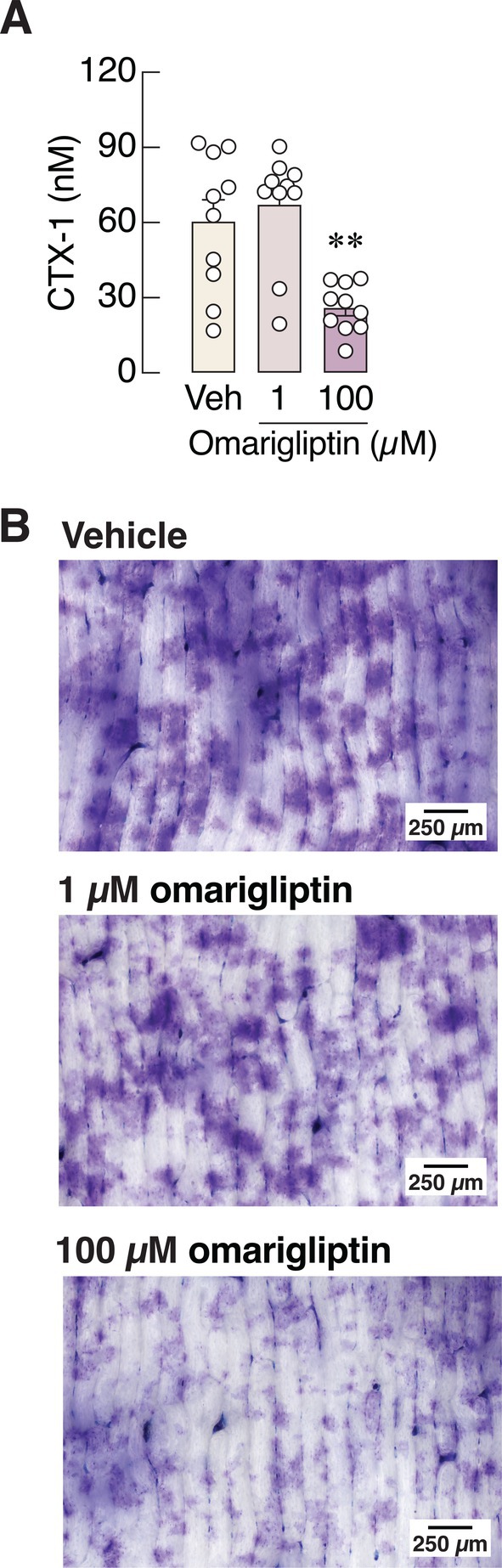
(A) Concentration of C‐terminal telopeptides of type I collagen (CTX‐1) in culture media obtained from bone marrow‐derived osteoclasts grown on bone slices treated with 1 and 100 μM omarigliptin. (B) representative photomicrographs of resorption pit stained with 1% toluidine blue in 1% sodium borate. Positive blue areas represent resorption pit created by osteoclasts. *n* = 10; **p < 0.01 vs. vehicle‐treated group by one‐way analysis of variance (ANOVA) with Dunnett's multiple comparisons test.

On the other hand, 100 μM omarigliptin—another potent DPP‐4 inhibitor—markedly reduced the number of TRAP‐positive cells at both the early differentiating and mature stages (Figure 6). The mRNA expression levels of various osteoclast markers (i.e., RANK, calcitonin receptor, TRAP, cathepsin K, integrin α_v_, integrin β_3_, V‐ATPase, and NFATc1) and osteoclast‐derived coupling factors [i.e., leukemia inhibitory factor (LIF) and cystatin C (CST3)] were significantly downregulated by 100 μM omarigliptin (Figure 7). We also assessed osteoclast activity by culturing M‐CSF‐ and RANKL‐stimulated bone marrow cells on cell‐free bone slices to mimic physiological conditions. The levels of CTX‐1, a bone resorption marker, released from bone slices were markedly decreased when primary osteoclasts were treated with 100 μM but not 1 μM omarigliptin (Figure 8). Microscopic examination revealed that 100 μM omarigliptin reduced toluidine blue staining of resorption pits on the bone slices (Figure 8). Thus, omarigliptin, but not vildagliptin, markedly suppressed osteoclastogenesis and osteoclast maturation.

### 3.4 DPP‐4 and Its Potential Substrates Were Locally Expressed in Osteoblasts and Osteoclasts

**FIGURE 9:**
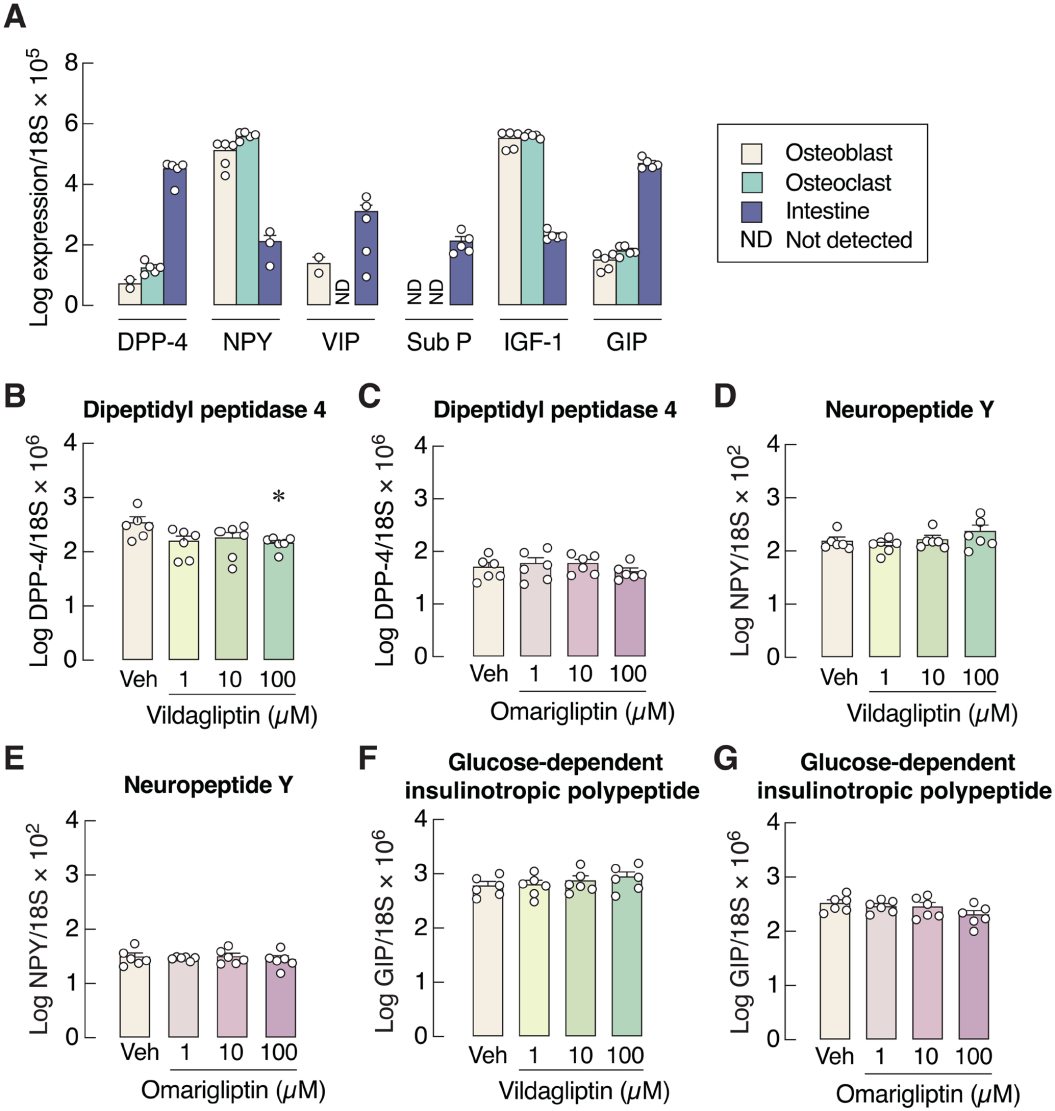
mRNA expression of (A) DPP‐4 and DPP‐4 substrates in primary osteoblasts, primary osteoclasts, and intestine (*n* = 5). mRNA expression of (B–C) DPP‐4, (D–E) neuropeptide Y (NPY), (F–G) glucose‐dependent insulinotropic polypeptide in primary osteoclasts after treatment with 1, 10, 100 μM vildagliptin or omarigliptin for 5 days. *n* = 6; *p < 0.05 vs. vehicle‐treated group by one‐way analysis of variance (ANOVA) with Dunnett's multiple comparisons test. ND, not detected.

Since DPP‐4 inhibitors directly affected bone cells, we further investigated whether these cells were able to express DPP‐4, which are the binding targets of vildagliptin and omarigliptin. We found that DPP‐4 was strongly expressed in primary osteoblasts and osteoclasts, although the expression levels were lower than those in the intestine (Figure 9). Potential DPP‐4 substrates, namely neuropeptide Y, IGF‐1, and GIP, were also expressed in both osteoblasts and osteoclasts. However, the expression levels of VIP and substance P were negligible in osteoclasts, making them unlikely to be substrates for bone cell‐derived DPP‐4. Vildagliptin (100 μM), but not omarigliptin, downregulated DPP‐4 expression in osteoclasts (Figure 9). However, the DPP‐4 mRNA expression was not correlated with the expression of osteoclast‐mediated bone resorption markers (Figure S2). Neither drug had an effect on the mRNA expression of neuropeptide Y or GIP in osteoclasts (Figure 9).

### 3.5 Vildagliptin and Omarigliptin Differentially Bound to the mDPP‐4 Protein, as Simulated by *In Silico* Molecular Docking and Molecular Dynamics

**FIGURE 10:**
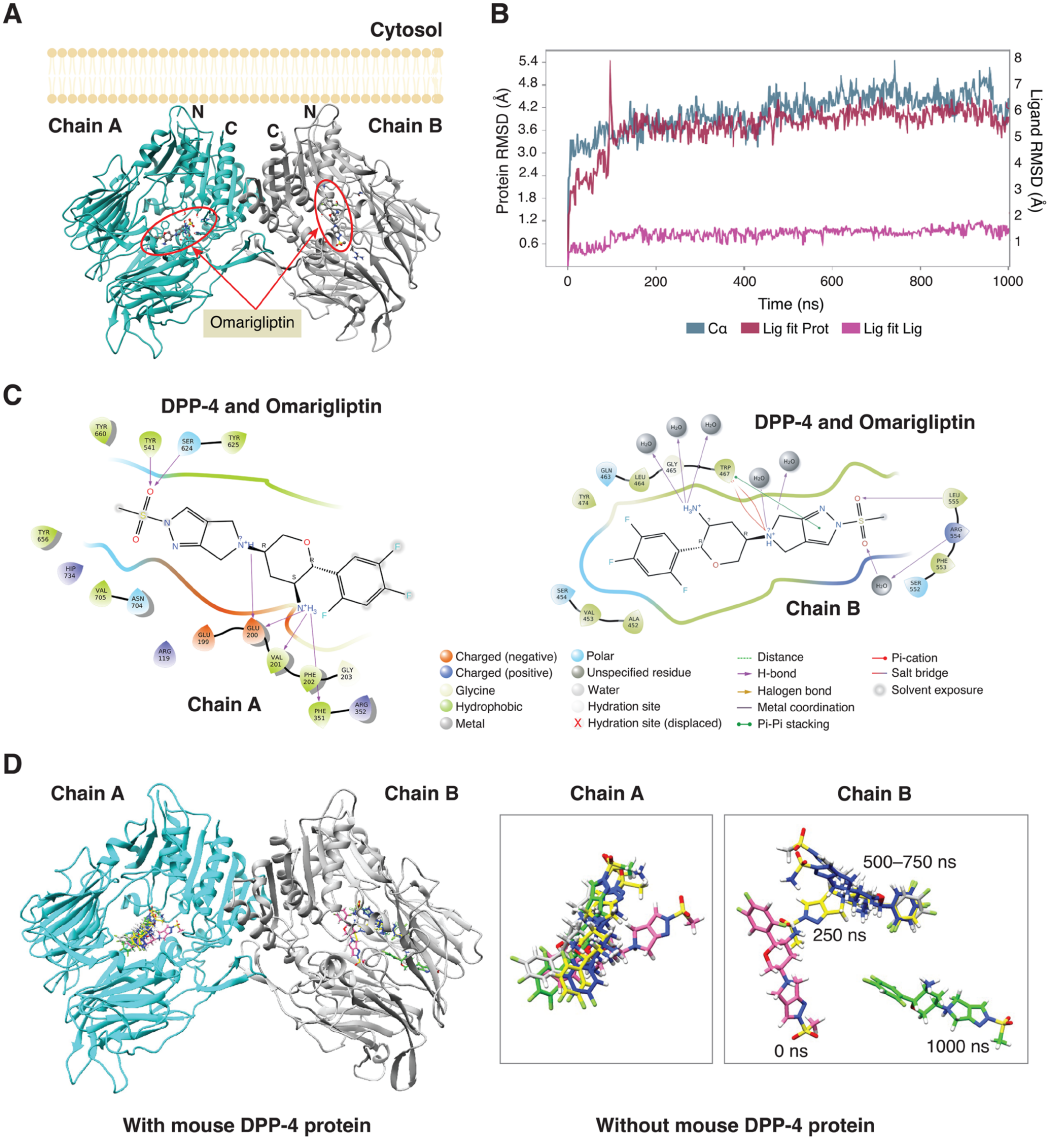
Molecular dynamics simulation depicting the binding of omarigliptin to mouse DPP‐4 dimers. (A) Two DPP‐4 subunits are shown in ribbons (cyan and gray) with omarigliptin in the active site of DPP‐4 dimers. The elements including carbon, nitrogen, oxygen, sulfur and fluoride are colored in gray, blue, red, yellow and cyan, respectively. (B) The Root Mean Square Deviation (RMSD) shows an atomic displacement of a ligand and protein throughout the simulation. (C) The 2D plot of protein‐ligand interaction visualized from molecular dynamics simulation at 1 ms. The diagrams depict the chemical interactions between the drug and DPP‐4 at above 30.0% of the simulation time. (D) The position of the drug is shown at different time points: 250 ns (yellow), 500 ns (blue), 750 ns (gray), and 1000 ns (green). The binding site of omarigliptin and DPP‐4 (chain B) shifts from its original position, indicating a gradual diffusion of omarigliptin over time.

The binding energy of omarigliptin in mice was predicted using the molecular docking tool, Autodock Vina. The docking score for mDPP‐4 was calculated to be −8.6 kcal/mol, which was comparable to the scores in rats (−8.0 kcal/mol, PDB: 2GBC) and humans (−8.4 kcal/mol, PDB: 4PNZ) (Table 2). Thereafter, the protein‐ligand complex of mDPP‐4 was subjected to molecular dynamics simulations for 1000 ns. The results clearly indicated that omarigliptin could bind to both subunits of the mDPP‐4 homodimer (Figure 10), but the dynamics of chain A and chain B were different. Specifically, the drug tightly bound to the mDPP‐4 dimer at the chain A subunit (i.e., stable binding). However, while it also bound to chain B, it showed slight diffusion from the original position as the simulation progressed beyond 50 ns (Figure 10 and Video S1). In chain A, omarigliptin was largely stabilized by the ionic interactions of Glu199 and Glu200 with the NH group of the drug. Additionally, Phe351 formed a Pi‐cation interaction with the aromatic ring moiety of omarigliptin (Figure 10). The root mean square deviation (RMSD) of omarigliptin and the protein backbone—representing the average difference in atomic positions—suggested a stable interaction between the drug and mDPP‐4 chain A (Figure 10). On the other hand, its interaction with chain B was mainly stabilized by hydrophobic interactions with nonpolar amino acids, including Ala452, Val453, Gly465, Leu464, and Tyr474 (Figure 10), suggesting a more loosely bound nature. Thus, it was apparent that omarigliptin exhibited tighter binding to one subunit of mDPP‐4 (chain A) with looser binding to the other subunit (chain B), as shown in Figure 10.

**TABLE 2:** Docking score of vildagliptin and omarigliptin to DPP‐4.

DPP‐4	Accession codes (PDB/AlphaFold)	Docking scores (kcal/mol)
Human		
Vildagliptin	3W2T	−7.1
Omarigliptin (MK‐3102)	4PNZ	−8.4
Rat		
Vildagliptin	2GBC	−6.7
Omarigliptin (MK‐3102)	2GBC	−8.0
Mouse		
Vildagliptin	AF‐P28843	−6.9
Omarigliptin (MK‐3102)	SWISS‐MODEL	−8.6

Unlike omarigliptin, DPP‐4 was found to tightly bind to vildagliptin through covalent bonding (Figures S3–S5). Specifically, as depicted in Figure S3, the cyanopyrrolidine moiety of vildagliptin forms a covalent bond with the amino acid Ser630 of human DPP‐4, which corresponds to Ser624 in mouse DPP‐4. The nitrile (C≡N) group of vildagliptin acts as a covalent trap, thereby reacting with the hydroxyl group (–OH) of Ser630 located in the active site of DPP‐4. Therefore, these two DPP‐4 inhibitors exhibit different protein‐ligand binding mechanisms, which might, in turn, affect cellular responses and the final outcome.

## 4 Discussion

It is well known that DM leads to a variety of detrimental outcomes on bone, including enhanced osteoclast‐mediated bone resorption, impaired osteoblast function, and even inappropriately low bone turnover, all of which result in poor bone quality, bone fragility and a high fracture risk in diabetic patients (Schwartz et al. 2001; Aeimlapa et al. 2019). In addition to the complications of DM itself, the use of certain antidiabetic agents may negatively affect bone integrity (Loke et al. 2009; Watts et al. 2016). For example, thiazolidinediones (e.g., pioglitazone) and sodium‐glucose co‐transporter 2 (SGLT2) inhibitors (e.g., canagliflozin) have been reported to induce bone loss (Habib et al. 2010; Watts et al. 2016). However, the presence of multiple factors in vivo—such as obesity and dyslipidemia—can complicate efforts to understand the underlying mechanisms of antidiabetic drugs on bone at the cellular level. In the present study, only 4 weeks of vildagliptin administration may not have been sufficient to alter visceral fat, although it was effective in improving lipid metabolism, as indicated by decreases in plasma total cholesterol and low‐density lipoprotein levels. Furthermore, we demonstrated the positive effects of DPP‐4 inhibitors, another class of antidiabetic drugs, on bone microstructure in vivo, as well as the direct actions of vildagliptin and omarigliptin on primary mouse osteoblasts and osteoclasts in vitro.

Previously, DPP‐4 inhibitors have been reported to exert beneficial effects on bone microarchitecture (Glorie et al. 2014; Charoenphandhu et al. 2018), presumably via prolonged activation of the incretin pathway, which eventually reduces plasma glucose levels and indirectly helps improve bone metabolism. The serum levels of GLP‐1 and GIP—both of which are incretins—were elevated in response to DPP‐4 inhibitor treatment (Mari et al. 2005; Farngren et al. 2012). Since GLP‐1 and GIP receptors are expressed in osteoblasts, osteoclasts, and osteocytes (Zhong et al. 2007; Pereira et al. 2015; Hansen et al. 2023), activation of these receptors likely plays a role in the regulation of bone turnover, which could help explain the positive effects of vildagliptin observed in vivo (Figure 1). In osteoclasts, GIP suppressed bone resorption by decreasing osteoclast marker expression and increasing osteoclast apoptosis. GIP also prevented osteoblast apoptosis without affecting the production of osteoclastogenic factors (Zhong et al. 2007; Hansen et al. 2023). Nevertheless, since both osteoblasts and osteoclasts can express DPP‐4, vildagliptin and omarigliptin likely exert direct actions on bone cells. These direct effects could be more beneficial to diabetic patients, given that strict glycemic control alone is often insufficient to completely restore diabetic osteopathy (Aeimlapa et al. 2018). In other words, we postulated that DPP‐4 inhibitors could benefit both glycemic control and bone metabolism.

Although the present study found that vildagliptin improved the trabecular bone microstructure of prediabetic rats (Figure 1), the underlying mechanisms of vildagliptin effects on bone at the cellular level remain largely unknown. Therefore, we conducted a comparative study between two types of DPP‐4 inhibitors, vildagliptin and omarigliptin, on bone cells in vitro. The study revealed that both vildagliptin and omarigliptin similarly increased the viability of primary mouse osteoblasts without altering markers of osteoblastic differentiation (Figure 2). Indeed, the effects of DPP‐4 inhibitors on osteoblast viability varied depending on the type and concentration of the inhibitors. In MC3T3‐E1 pre‐osteoblast‐like cells, 20 μM omarigliptin did not affect cell viability but promoted osteoblast differentiation and mineralization via the p38/Akt pathway. High concentrations of DPP‐4 inhibitors, such as 250–500 μM trelagliptin or 100–500 μM omarigliptin, decreased pre‐osteoblastic cell viability when the cells were exposed to these drugs for 14 days (Liao et al. 2021; Shao et al. 2021). Furthermore, DPP‐4 inhibitors also promoted osteoblast differentiation and mineralization through distinct intracellular mechanisms. Trelagliptin was able to enhance pre‐osteoblastic MC3T3‐E1 cell differentiation and mineralization by activating the AMP‐activated protein kinase α pathway (Shao et al. 2021), whereas the osteogenic effect of anagliptin was mediated via the Wnt signaling pathway (Dong et al. 2020). On the other hand, saxagliptin decreased osteocyte density and osteoblast number in rats by promoting adipogenesis while attenuating the osteotropic differentiation of mesenchymal stem cells (Sbaraglini et al. 2014). We, therefore, hypothesize that the diverse intracellular signaling pathways and final outcomes of various DPP‐4 inhibitors likely result from the binding pattern, kinetics, or affinity of each DPP‐4 inhibitor to the DPP‐4 homodimer on target cells.

How DPP‐4 inhibitors increase viability, prolong survival, or reduce apoptosis of primary osteoblasts requires further investigation. Previously, He et al. (2024) found that vildagliptin reduced osteoblast apoptosis, as determined by flow cytometry. In the present study, we performed an MTT assay after the cells had been allowed to proliferate for 72 h, and then were exposed to vildagliptin or omarigliptin for 5 days. Since the cells were expected to have already exited the proliferative phase, our PCR experiments showed that neither vildagliptin nor omarigliptin, at this stage, altered the transcription of genes related to cell cycle regulation, namely cyclin D1, *Cdkn1a*, or *Cdkn2a* (Figure S1). Furthermore, DPP‐4 inhibitors have been reported to accelerate osteoblastic differentiation in a Runx2‐dependent manner (Liao et al. 2021). While the accelerated differentiation of osteoblasts may initially increase the number of mature osteoblasts, it can also trigger intrinsic apoptosis, as indicated by the downregulation of Bcl‐2 expression. As shown in Figure S1, low‐dose vildagliptin slightly downregulated Bcl‐2 expression, consistent with the initiation of apoptosis following accelerated differentiation, whereas its higher doses as well as all doses of omarigliptin did not alter Bcl‐2 expression, suggesting that intrinsic apoptosis was not induced and that mature osteoblasts could maintain their survival.

We further demonstrated in primary osteoclast culture that vildagliptin did not suppress osteoclastogenesis—as indicated by the unchanged number of TRAP‐positive cells (Figure 4)—and only modestly altered the expression levels of bone resorption markers (Figure 5). In contrast, omarigliptin potently suppressed osteoclastogenesis and markedly downregulated the expression of genes related to osteoclast differentiation (e.g., RANK and calcitonin receptor) and resorptive activities (e.g., NFATc1, TRAP, cathepsin K, V‐ATPase) (Figure 7). Although 100 μM vildagliptin may eventually enhance the functions of integrin α_v_ (Figure 5E) and V‐ATPase (Figure 5G), it may not fully induce osteoclastic resorption without the upregulation of cathepsin K and TRAP expression. The exact reason why vildagliptin and omarigliptin exert differential effects on integrin and V‐ATPase expression remains unclear. Despite being DPP‐4 inhibitors, their chemical structures and binding profiles differ substantially. *In silico* molecular docking in this study revealed that vildagliptin and omarigliptin interact with DPP‐4 in distinct ways. Therefore, the observed differences in their effects may reflect differences in binding affinity or kinetics. Since DPP‐4 inhibition could reduce osteoclast formation and bone resorption, future knockdown and overexpression experiments may help decipher the underlying cellular signaling pathways, such as the RANKL and TNF‐α pathways, both of which are essential for osteoclastogenesis.

The direct actions of both drugs on osteoclasts were supported by the presence of DPP‐4 transcripts in mouse multinucleated osteoclasts (Figure 9). Generally, DPP‐4 and its substrates are expressed by various cell types, such as enterocytes and endothelial cells (Darmoul et al. 1992; Liu et al. 2024) (for a review, please see Glorie et al. 2016). The functional roles of DPP‐4 and its outcomes are likely tissue‐specific. For instance, endothelial DPP‐4 is involved in the regulation of plasma glucose levels through the cleavage of both GIP and GLP‐1 molecules. However, hematopoietic cell‐derived DPP‐4 selectively inactivates only GIP (Mulvihill et al. 2017). In general, DPP‐4 expression levels often correlate with DPP‐4 activity (Chowdhury et al. 2016; Mulvihill et al. 2017). However, the DPP‐4 protein levels and enzymatic activity are not correlated in certain tissues, such as bone marrow, liver, and spleen (Baggio et al. 2020). Since DPP‐4 was found to be strongly expressed in monocytes (Ellingsen et al. 2007), which serve as osteoclast precursors, this partially explains why omarigliptin exerted more potent effects on osteoclasts than on osteoblasts.

In the basic multicellular unit, several cell types, including osteoclasts, osteoblasts, osteocytes, bone‐lining cells, osteomacs (bone macrophages), and vascular endothelial cells, orchestrate the bone remodeling process (Kular et al. 2012). DPP‐4 is also expressed in vascular endothelial cells and immune cells (Mulvihill et al. 2017; Rao et al. 2019), which may contribute to the beneficial effects of DPP‐4 inhibitors on bone microstructure. For instance, DPP‐4 inhibitors have been shown to shift macrophage polarization from the pro‐inflammatory M1 type toward the anti‐inflammatory M2 type (Zhuge et al. 2016; De Nigris et al. 2021), and to reduce the release of macrophage‐derived pro‐inflammatory cytokines, which might, in turn, decrease bone resorption (Ishida et al. 2019). Furthermore, DPP‐4 also exerts non‐enzymatic functions, including roles in cell communication and cell adhesion. For example, osteoclasts have been shown to express and release DPP‐4 protein to regulate glucose metabolism (Weivoda et al. 2020).

Indeed, the activities of osteoclasts and osteoblasts must be coupled to maintain normal bone remodeling in vivo. Osteoclasts also secrete several coupling factors, such as LIF and cystatin C, to ensure that bone resorption is always followed by bone formation (Danjo et al. 2007; Borggaard et al. 2022). Thus, targeted inhibition of bone resorption without appropriate fine‐tuning of osteoblast‐mediated bone formation can eventually lead to unfavorable effects on bone mass (Weivoda et al. 2020). Although omarigliptin suppressed LIF and CST3 mRNA expression in primary osteoclasts (Figure 7), it also increased osteoblast viability in vitro (Figure 2). Therefore, unlike vildagliptin, omarigliptin could be a promising drug, as it attenuates osteoclast‐mediated bone resorption while concurrently increasing bone formation.

In monocyte/macrophage‐derived osteoclasts, DPP‐4/CD26 expression is progressively upregulated under certain pathological conditions, such as osteolytic bone lesions from multiple myeloma, osteosarcoma, adenocarcinoma (Nishida et al. 2014). Inhibition of osteoclastic CD26 by a monoclonal antibody against CD26 hindered RANKL‐induced osteoclast differentiation through the p38/MAPK pathway, particularly during early osteoclast differentiation (Nishida et al. 2014). However, consistent with our findings, Nishida et al. (2014) reported that vildagliptin had no direct effect on human osteoclast‐mediated bone resorption in vitro. Although 100 μM vildagliptin suppressed DPP‐4 expression in osteoclasts, the downregulation of DPP‐4 mRNA expression was not correlated with the expression of osteoclast‐mediated bone resorption markers (Figure S2). Additionally, other DPP‐4 inhibitors, for example, linagliptin, did not attenuate RANKL‐, TNF‐α‐, or lipopolysaccharide (LPS)‐induced osteoclastogenesis in vitro, although subcutaneous injection of linagliptin effectively suppressed the expression of osteoclast markers and bone resorption in LPS‐treated mice (Ishida et al. 2019). Linagliptin could also directly decrease the number of TRAP‐positive osteoclasts as well as the expression of osteoclast markers without changing DPP‐4 expression (Lee et al. 2025). Sitagliptin, on the other hand, was able to suppress osteoclastic bone resorption in estrogen‐deficient rats as well as RANKL‐induced osteoclastogenesis in vitro (Wang et al. 2017). These findings support our hypothesis that the eventual outcomes of DPP‐4 inhibitors on osteoclastogenesis and resorptive activity depend on various factors, including chemical structure, drug‐protein dynamics, binding energy (as indicated by the Vina score) or kinetics, and the presence of covalent bonding (as in the case of vildagliptin). The use of a monoclonal antibody against the extracellular portion of DPP‐4 may result in different outcomes compared to small molecules.

Further *in silico* analyzes using molecular docking and molecular dynamics suggested that the distinct actions of vildagliptin versus omarigliptin on osteoclasts may be related to their chemical structures and binding dynamics. Omarigliptin was found to interact more effectively with the DPP‐4 homodimer than vildagliptin due to a larger area of interaction via non‐covalent bonding between the DPP‐4 protein and omarigliptin. Additionally, the greater potency of omarigliptin in suppressing osteoclastogenesis and osteoclast activity might be associated with its binding pattern to DPP‐4 active sites. Previous comparative binding analyzes showed that omarigliptin binds to the S1, S2, and S2 extensive subsites of DPP‐4, whereas vildagliptin binds primarily to the S1 and S2 subsites (Nabeno et al. 2013; Nojima et al. 2016). Furthermore, the inhibitory capability of DPP‐4 inhibitors may be related to their binding affinity to DPP‐4. Analysis of DPP‐4 protein–DPP‐4 inhibitor interactions using the quantum mechanical fragment molecular orbitals method revealed that a greater contact area and stronger hydrophobic interaction energy with DPP‐4 active sites contributed to a higher degree of inhibitory activity (Arulmozhiraja et al. 2016). In the present study, we also found a loose hydrophobic interaction between chain B of the DPP‐4 homodimer and omarigliptin, alongside a tight ionic interaction between chain A and omarigliptin (Figure 10). Meanwhile, additional binding to the S2 extensive subsite, an oscillatory hydrophobic interaction between omarigliptin and the DPP‐4 protein, and the presence of covalent bonding between the DPP‐4 protein and vildagliptin may also contribute to the differential effects of omarigliptin and vildagliptin in regulating osteoclastic bone resorption.

However, the cellular and molecular mechanisms of DPP‐4‐induced osteoclastogenesis and the exact DPP‐4 substrates in the local milieu remain unclear and require further investigation. Although DPP‐4 and some DPP‐4 substrates are locally expressed by both osteoblasts and osteoclasts, their functional roles in bone metabolism are largely unknown. DPP‐4 is able to exert biological functions through both catalytic and non‐catalytic activities. The cleavage of circulating active GIP and GLP‐1 is the well‐known catalytic activity of DPP‐4, while the non‐catalytic activity of membrane‐bound DPP‐4/CD26 on T‐cells involves direct interaction with caveolin‐1 on antigen‐presenting cells (APCs), leading to T‐cell proliferation and CD86 expression in APCs (Ohnuma et al. 2006). Since inhibition of DPP‐4 activity enhances the transcription of DPP‐4 substrate genes, particularly in human endothelial progenitor cells (EPCs) (Liu et al. 2016), our findings that showed unchanged mRNA levels of DPP‐4 substrates, namely neuropeptide Y and GIP, after exposure to DPP‐4 inhibitors suggest that both osteoclast‐derived DPP‐4 substrates are unlikely to be involved in the DPP‐4 signaling cascade.

Nevertheless, the limitations of the present study include the absence of certain static and dynamic bone histomorphometric data, for example, osteoblast number (N.Ob/BS), osteoclast number (N.Oc/BS), osteoclast surface (Oc.S/BS), and bone formation rate (BFR/BS). A larger sample size may be required to demonstrate a statistically significant change in BV/TV. In addition, we selected three concentrations of DPP‐4 inhibitors, that is, 1, 10, and 100 μM for dose–response studies, based on the fact that moderate‐to‐high concentrations (10–100 μM) are often required to demonstrate effects on bone cells in vitro. For example, the number of TRAP‐positive multinucleated osteoclasts, mRNA expression of osteoclast‐specific genes, and resorptive activity were markedly decreased by ~30–120 μM sitagliptin (Wang et al. 2017). Although it is not surprising to observe the effects of DPP‐4 inhibitors mainly at a high dose of 100 μM, a limitation exists in the dose–response range between 10 and 100 μM. Lastly, it remains unclear how vildagliptin‐induced downregulation of DPP‐4 expression affects osteoclast‐mediated bone resorption. Future investigations using various monoclonal antibodies against DPP‐4, site‐directed mutagenesis, or siRNA knockdown, and direct measurement of DPP‐4 activity in vildagliptin‐ or omarigliptin‐treated osteoclasts may help elucidate the exact roles of DPP‐4 in osteoclasts. Since most studies have employed knockdown or overexpression approaches in osteoclast precursors (mononuclear cells) rather than multinucleated mature osteoclasts (Mulvihill et al. 2017; Zhou et al. 2026), there is a technical limitation in determining the direct effects of DPP‐4 activity on multinucleated osteoclast‐mediated bone resorption.

In conclusion, the DPP‐4 inhibitors vildagliptin and omarigliptin exerted differential effects on bone cells. Although vildagliptin improved the bone microstructure of prediabetic rats, it was unable to downregulate osteoclastogenesis or the expression of key osteoclast transcripts. It is possible that vildagliptin predominantly affects osteoblasts by prolonging their survival, thereby increasing their viability after 5 days of exposure. This would allow more time for osteoblasts to produce matrix proteins and facilitate mineralization. In contrast, omarigliptin effectively attenuated osteoclastic bone resorption by suppressing osteoclast differentiation, maturation, and resorptive activity. Omarigliptin might, therefore, be a preferable DPP‐4 inhibitor to help decelerate bone loss in diabetic patients. However, further investigations are required to elucidate the cellular and molecular mechanisms of each DPP‐4 inhibitor, particularly omarigliptin, on bone turnover in vivo. Since patients with T1DM and T2DM differently manifest a variety of bone defects—for example, osteopenia (particularly in T1DM), impaired bone strength, and fragility fractures—our findings provide a foundation for drug discovery targeting both DM and bone cells, potentially leading to better bone health for prediabetic and diabetic patients.

## Author Contributions

R.A., J.P., J. Teerapornpuntakit, N.P., K.W., S.S., S.C., N. Chattipakorn, N. Charoenphandhu conceived and designed research. R.A., J. Thongbunchoo, N.A., P.S. performed experiments. R.A., J.P., J. Teerapornpuntakit, N.P., K.W., S.S., S.C., N. Chattipakorn, N. Charoenphandhu analyzed data. R.A., J.P., J. Teerapornpuntakit, N.P., K.W., S.S., S.C., N. Chattipakorn, N. Charoenphandhu interpreted results of experiments. R.A., J.P., J. Teerapornpuntakit, N. Charoenphandhu prepared figures. R.A., J.P., J. Teerapornpuntakit, N.P., K.W., S.S., S.C., N. Chattipakorn, N. Charoenphandhu drafted manuscript. R.A., J.P., J. Teerapornpuntakit, N.P., K.W., S.S., S.C., N. Chattipakorn, N. Charoenphandhu criticized, discussed, concluded the findings, edited and revised manuscript. All authors approved final version of manuscript.

## Funding

This work was supported by Mahidol University (to R. Aeimlapa). Fundamental Fund through Mahidol University [fiscal year 2024–2026 by National Science Research and Innovation Fund (NSRF); to N. Charoenphandhu], Science and Technology Development Fund by NSRF (ST; to N. Charoenphandhu), Central Instrumental Facility (CIF/CNI), Faculty of Science, Mahidol University (to R. Aeimlapa and N. Charoenphandhu), Research Assistant Grant from Faculty of Science, Mahidol University (to J. Thongbunchoo), Research Cluster Development Fund, Mahidol University (to N. Charoenphandhu), the NSRF via the Program Management Unit for Human Resources & Institutional Development, Research and Innovation (PMU‐B) (Grants B11F670112 and B11F680022; to N. Charoenphandhu), Burapha University‐TSRI‐NSRF (Fundamental Fund; fiscal year 2025; to K. Wongdee). The equipment of this research project is supported by Faculty of Science, Mahidol University (to R. Aeimlapa).

## Ethics Statement

All experimental protocols were approved by the Institutional Animal Care and Use Committee (IACUC) of the Faculty of Medicine, Chiang Mai University, and the Faculty of Science, Mahidol University.

## Conflicts of Interest

The authors declare no conflicts of interest.

## Supporting information


**Figures S1‐S5:** cph470103‐sup‐0001‐FiguresS1‐S5.pdf.

**Table S1:** cph470103‐sup‐0002‐TableS1.pdf. *Mus musculus* primers for real‐time PCR.

**Video S1:** The molecular dynamics simulations of DPP‐4 and omarigliptin. The molecular dynamics was simulated for a duration of 1 ms using the NPT ensemble and OPLS_2005 force field. The two subunits of DPP‐4 dimer are shown in ribbons (chain A, red; chain B, yellow). The drugs are shown in sticks (carbon, nitrogen, oxygen, sulfur and fluoride are colored in gray, blue, red, yellow and cyan, respectively).

## Data Availability

The data that support the findings of this study are available from the corresponding author upon reasonable request.
