# Supplementary material for: Vildagliptin and Omarigliptin Differentially Bind to DPP‐4 Homodimers and Modulate Osteoclast‐Mediated Bone Resorption
Source: Compr Physiol. 2026 Jan 21;16(1):e70103. doi: 10.1002/cph4.70103 (PMC12824438; doi:10.1002/cph4.70103)
Supplement: Supplementary file 1 — Figures S1‐S5: cph470103‐sup‐0001‐FiguresS1‐S5.pdf. [file CPH4-16-e70103-s001.pdf]

Supplementary Figures S1–S5

ORIGINAL ARTICLE

Vildagliptin and omarigliptin differentially bind to DPP-4 homodimers and modulate osteoclast-mediated bone resorption

Ratchaneevan Aeimlapa[1,2], Jiraporn Panmanee[3], Jarinthorn Teerapornpuntakit[1,4], Kannikar Wongdee[1,5], Jirawan Thongbunchoo[1,2], Nattapon Panupinthu[1,2], Saovaros Svasti[6], Nattayaporn Apaijai[7,8], Piangkwan Sa-nguanmoo[7,9], Siriporn Chattipakorn[7,10], Nipon Chattipakorn[7,8,11], Narattaphol Charoenphandhu[1,2,11,12] *

[1] Center of Calcium and Bone Research (COCAB), Faculty of Science, Mahidol University, Bangkok, Thailand
[2] Department of Physiology, Faculty of Science, Mahidol University, Bangkok, Thailand
[3] Research Center for Neuroscience, Institute of Molecular Biosciences, Mahidol University, Nakhon Pathom, Thailand
[4] Physiology Division, Preclinical Science, Faculty of Medicine, Thammasat University, Pathum Thani, Thailand
[5] Faculty of Allied Health Sciences, Burapha University, Chonburi, Thailand
[6] Thalassemia Research Center, Institute of Molecular Biosciences, Mahidol University, Nakhon Pathom, Thailand
[7] Cardiac Electrophysiology Research and Training Center, Faculty of Medicine, Chiang Mai University, Chiang Mai, Thailand
[8] Department of Physiology, Faculty of Medicine, Chiang Mai University, Chiang Mai, Thailand
[9] Department of Physical Therapy, Faculty of Associated Medical Sciences, Chiang Mai University, Chiang Mai, Thailand
[10] Department of Oral Biology and Diagnostic Sciences, Faculty of Dentistry, Chiang Mai University, Chiang Mai, Thailand
[11] The Academy of Science, The Royal Society of Thailand, Bangkok, Thailand
[12] Institute of Molecular Biosciences, Mahidol University, Nakhon Pathom, Thailand

Running title: DPP-4 inhibitors modulate osteoclast activities

*** Corresponding author**

Narattaphol Charoenphandhu, M.D., Ph.D.
Department of Physiology
Faculty of Science, Mahidol University
Rama VI Road, Bangkok 10400
Thailand
E-mail: naratt@narattsys.com

Keywords: bone histomorphometry; bone loss; diabetes mellitus; dipeptidyl peptidase-4 (DPP-4) inhibitor; holotomography; *in silico* molecular dynamics; osteoblast

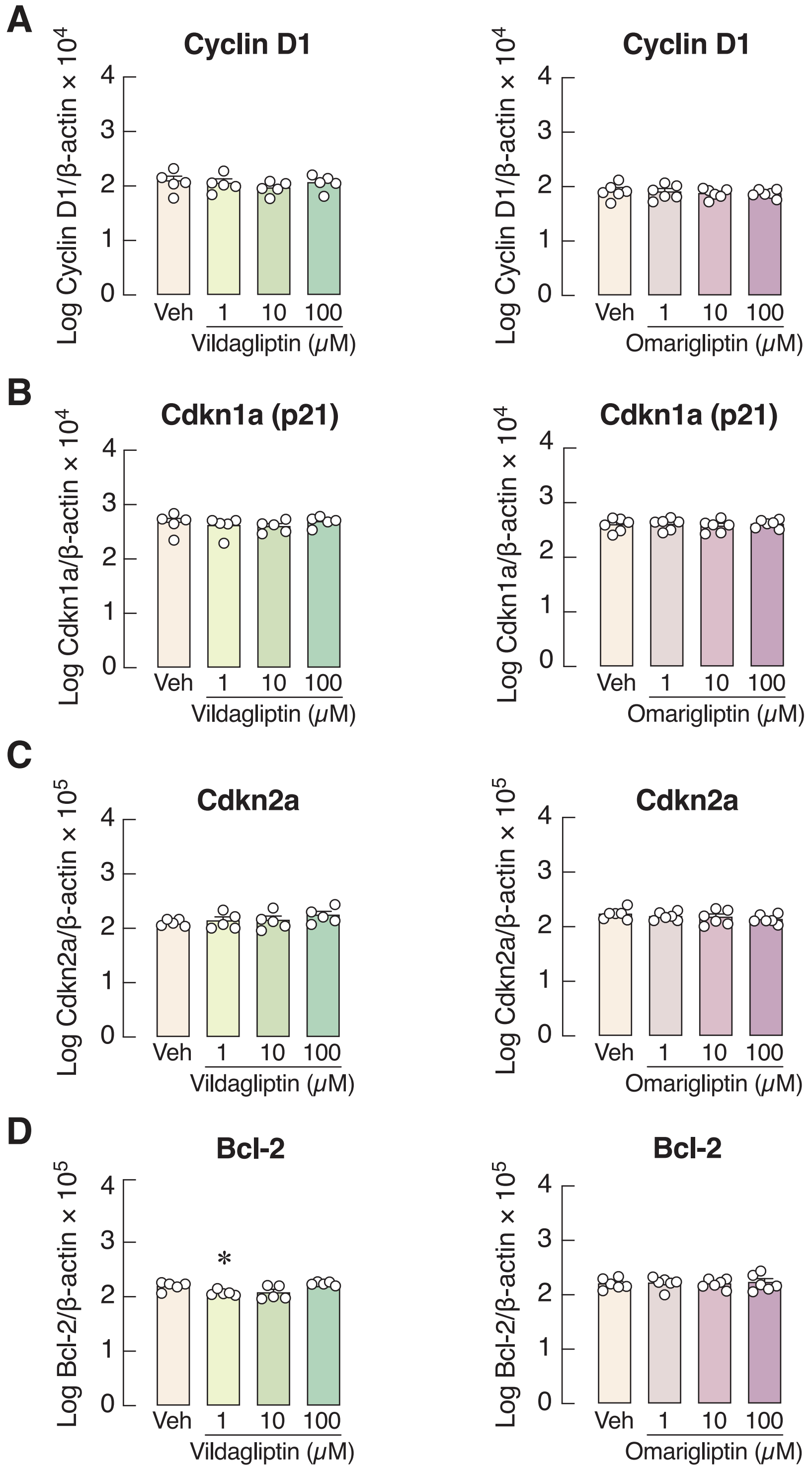

Figure S1: Aeimlapa et al.

Figure S1: mRNA expression levels of three cell cycle-related genes, namely (A) cyclin D1, (B) cyclin-dependent kinase inhibitor 1A (Cdkn1a; p21), (C) cyclin-dependent kinase inhibitor 2A (Cdkn2a), and (D) a key regulator of apoptosis Bcl-2 in primary osteoblasts after treated with 1, 10, 100 μM vildagliptin or omarigliptin for 5 days (n = 5–6). *P < 0.05 vs. vehicle-treated group (Veh) by one-way analysis of variance (ANOVA) with Dunnett's multiple comparison test.

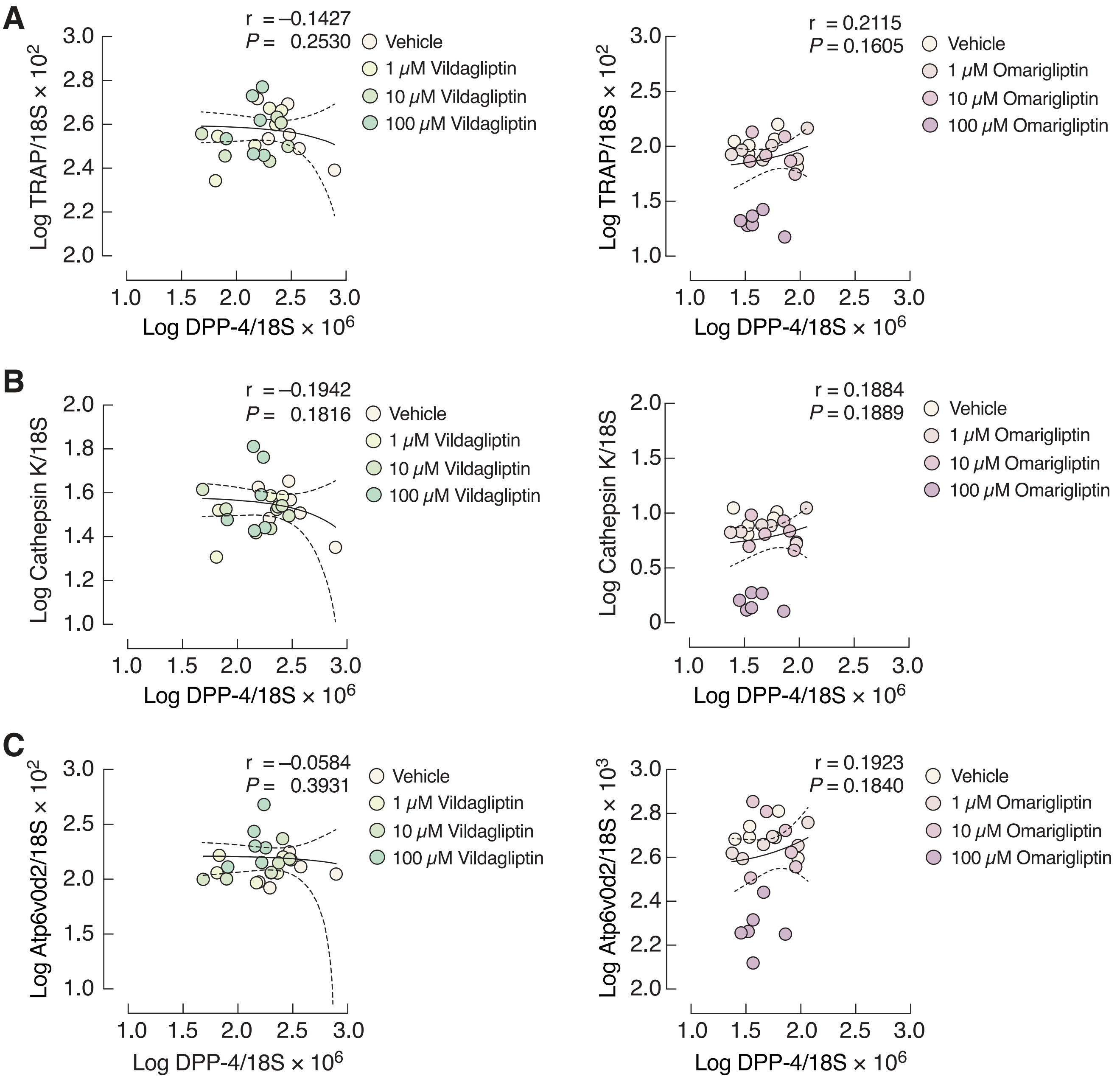

Figure S2: Aeimlapa et al.

Figure S2: Correlation plots between mRNA expressions of osteoclast-derived DPP-4 and (A) tartrate-resistant acid phosphatase (TRAP), (B) cathepsin K, (C) vacuolar ATPase H^+ Transporting V0 Subunit D2 (Atp6v0d2 or V-ATPase) in primary osteoclasts after treated with 1, 10, 100 μM vildagliptin or omarigliptin for 5 days (n = 6). Dashed line represents the 95% confidence bands of the best-fit line. r, Pearson correlation coefficient. P, p-value of the correlation.

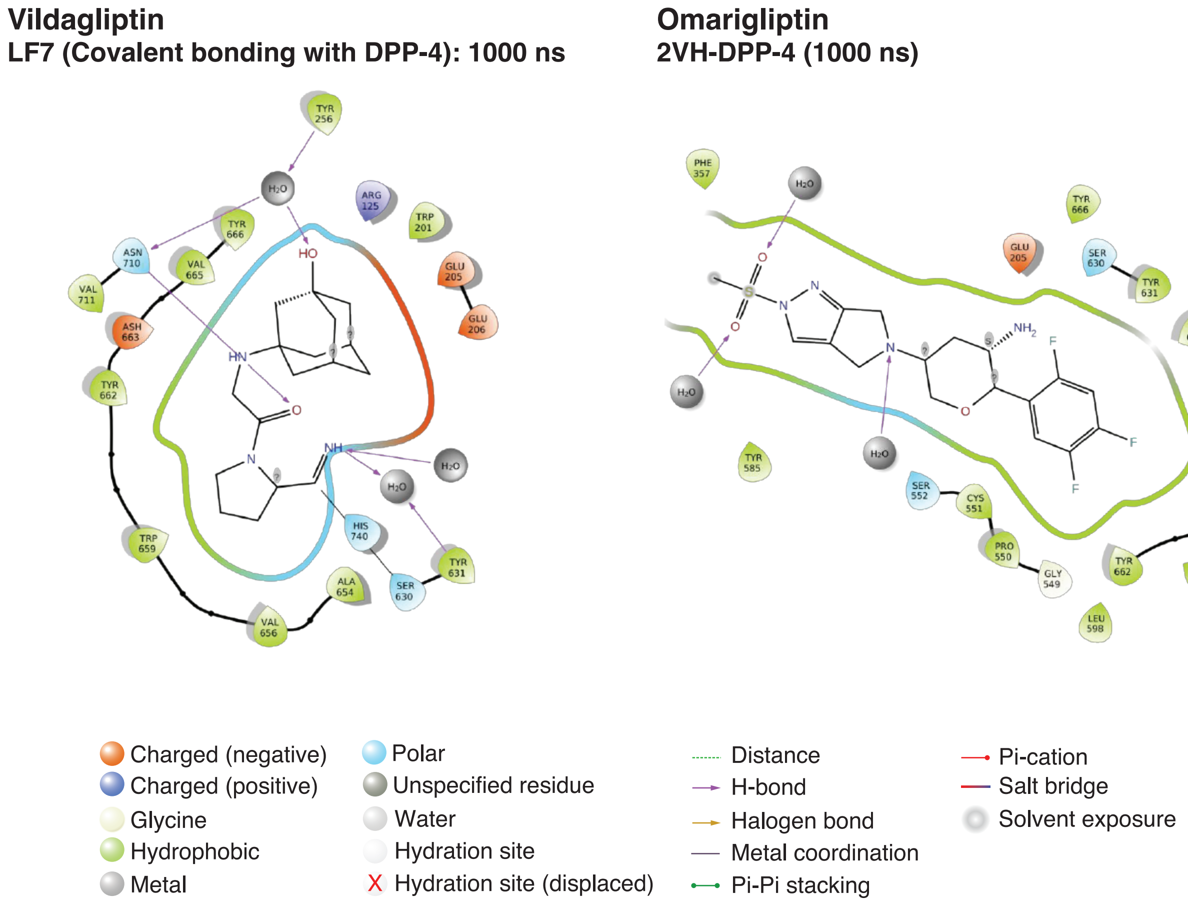

Figure S3: Aeimlapa et al.

Figure S3: The 2D plot of protein-ligand interaction visualized from molecular dynamics simulation at 1 ms of human DPP-4 complexes with omarigliptin (PDB: 4PNZ) and vildagliptin (PDB: 3W2T).

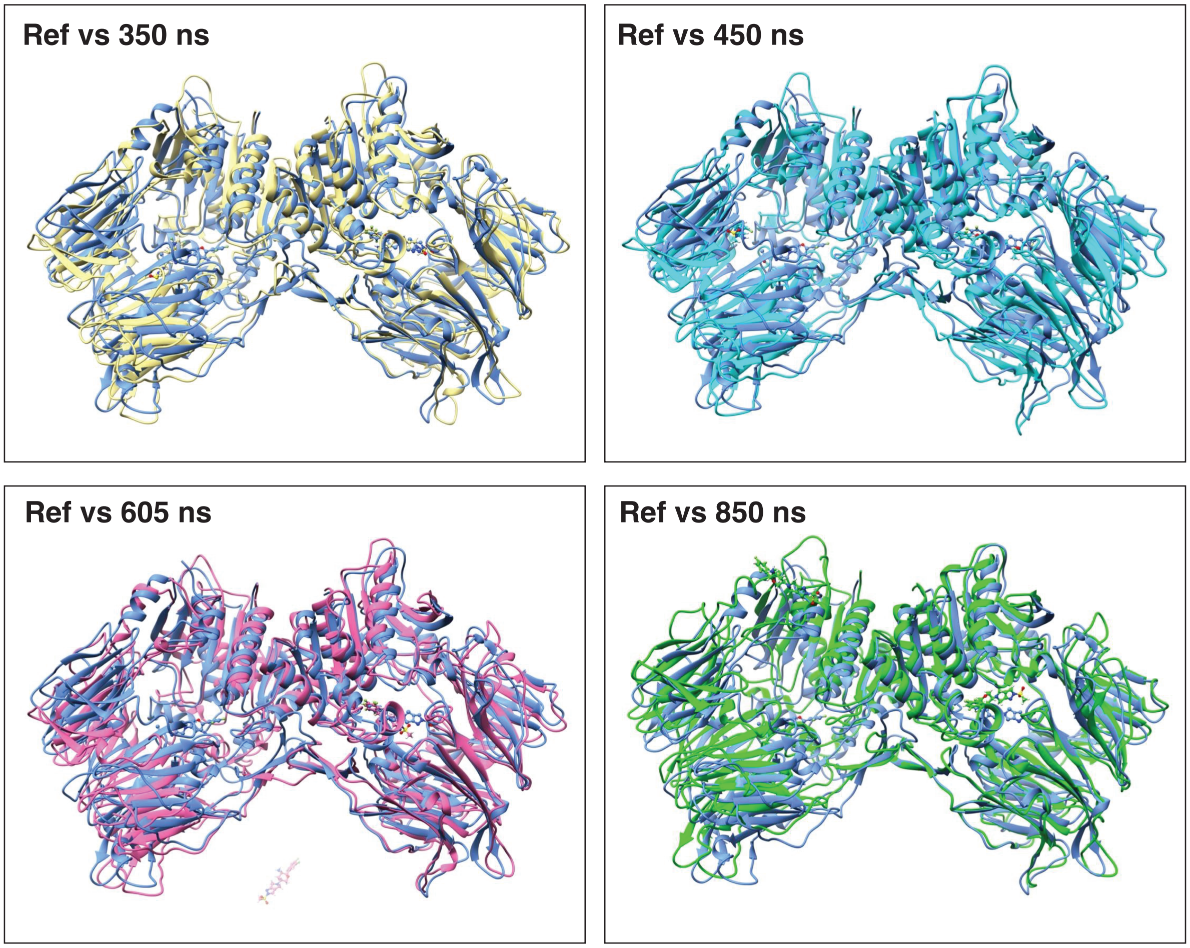

Figure S4: Aeimlapa et al.

Figure S4: Molecular dynamics simulation at 1 ms of human DPP-4 complexes with omarigliptin (PDB: 4PNZ). The position of the drug is shown at different time points: 350 ns (yellow), 450 ns (cyan), 605 ns (pink), and 850 ns (green). The protein reference (at 0 ns) is shown in ribbons (cyan). The proteins at different time points are shown in ribbons as follows; 350 ns (yellow), 450 ns (cyan), 605 ns (pink), and 850 ns (green).

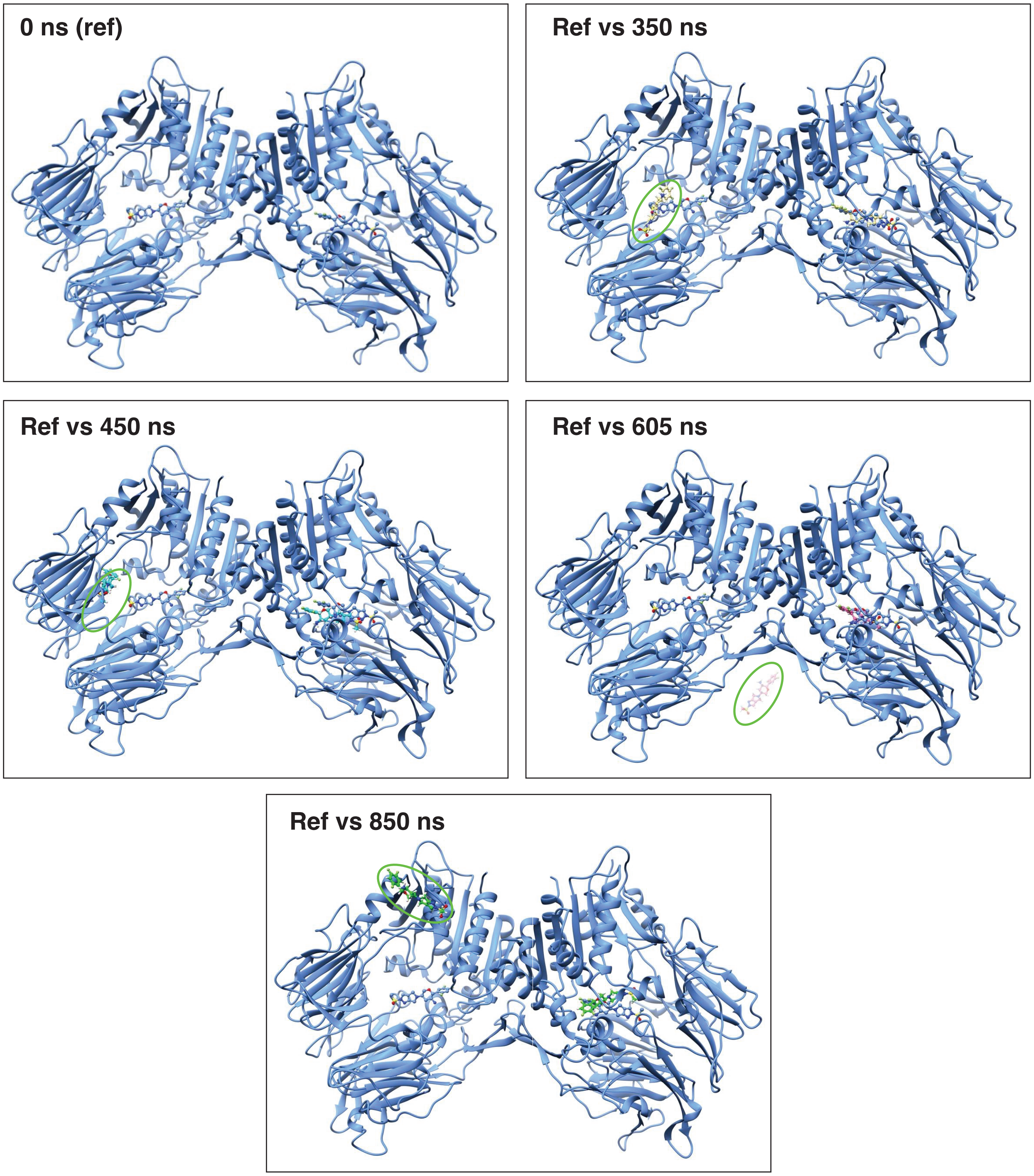

Figure S5: Aeimlapa et al.

Figure S5: Molecular dynamics simulation at 1 ms of human DPP-4 complexes with omarigliptin (PDB: 4PNZ). The position of the drug is shown at different time points: 350 ns (yellow), 450 ns (cyan), 605 ns (pink), and 850 ns (green). The protein reference (at 0 ns) is shown in ribbons.
