# Supplementary material for: Vildagliptin and Omarigliptin Differentially Bind to DPP‐4 Homodimers and Modulate Osteoclast‐Mediated Bone Resorption
Source: Compr Physiol. 2026 Jan 21;16(1):e70103. doi: 10.1002/cph4.70103 (PMC12824438; doi:10.1002/cph4.70103)
Supplement: Supplementary file 2 — Table S1: cph470103‐sup‐0002‐TableS1.pdf. Mus musculus primers for real‐time PCR. [file CPH4-16-e70103-s003.pdf]

Supplemental Table S1. *Mus musculus* primers for real-time PCR

Gene	Accession no.	Primer (Forward/Reverse)	Product size (bp)	Annealing temp. (°C)
Osteoblast differentiation markers				
Runx2	NM_001146038.2	5′-AGGGACTATGGCGTCAAACA-3′<br>5′-GGCTCACGTCGCTCATCTT-3′	137	57
ALP	NM_001287172.1	5′-TCTCCAGACCCTGCAACCTC-3′<br>5′-CATCCTGAGCAGACCTGGTC-3′	150	58
Osteocalcin	NM_001305448.1	5′-ACGGTATCACTATTTAGGACCTGTG-3′<br>5′-ACTTTATTTTGGAGCTGCTGTGAC-3′	141	57
Osteoblast-derived osteoclastogenic factors				
RANKL	NM_011613.3	5′-CGCTCTGTTCCTGTACTTTCG-3′<br>5′-CTCTCCAGAGTCGAGTCCTGC-3′	126	57
M-CSF	NM_007778.4	5′-GACCCTCGAGTCAACAGAGC-3′<br>5′-TGTCAGTCTCTGCCTGGATG-3′	236	58
Osteoclast differentiation markers				
RANK	NM_009399.3	5′-CATCTTCGGCGTTTACTACAGG-3′<br>5′-TCCACTTAGACTACTGCAAGCA-3′	91	57
Calr	NM_001355192.1	5′-CGGACTTTGACACAGCAGAA-3′<br>5′-GTCACCCTCTGGCAGCTAAG-3′	247	55
TRAP	NM_001102405.1	5′-CACTCCCACCCTGAGATTTGT-3′<br>5′-CATCGTCTGCACGGTTCTG-3′	118	57
Cathepsin K	NM_007802.4	5′-GAACGAGAAAGCCCTGAAGAGA-3′<br>5′-TATCGAGTGCTTGCTTCCCTTC-3′	190	57
Integrin α_v	NM_008402.3	5′-GGGTGATCATCTTGGCAGTT-3′<br>5′-GAACTTGGAGCGGACAGAAG-3′	205	58
Integrin β_3	NM_016780.2	5′-GCTCATTGGCCTTGCTACTC-3′<br>5′-CCCGGTAGGTGATATTGGTG-3′	167	58
Atp6v0d2	NM_175406.3	5′-GACCCTGTGGCACTTTTTGT-3′<br>5′-GTGTTTGAGCTTGGGGAGAA-3′	102	57
c-Fos	NM_010234.2	5′-CCAGTCAAGAGCATCAGCAA-3′<br>5′-AAGTAGTGCAGCCCGGAGTA-3′	247	55
NFATc1	NM_001164112.1	5′-CCGTTGCTTCCAGAAAATAACA-3′<br>5′-TGTGGGATGTGAACTCGGAA-3′	152	54
DPP4 and DPP4 substrates				
DPP4	NM_010074.3	5′-GTGGCAAGAGGGGATCACTA-3′<br>5′-CCCAGCCTGTGGTACTCATT-3′	184	60
NPY	NM_023456.3	5′-TGGACTGACCCTCGCTCTAT-3′<br>5′-TGTCTCAGGGCTGGATCTCT-3′	187	60
VIP	NM_001313969.1	5′-CTAGCCAGCTACAGCCAACC-3′<br>5′-ATCAGGAATGCCAGGAACTG-3′	186	59
Sub P	NM_001311060.2	5′-GTCTGACCGCAAAATCGAAC-3′<br>5′-CATTGCCTCCTTGATCTGGT-3′	152	56
IGF-1	NM_010512.5	5′-CTGGTGGATGCTCTTCAGTTCG-3′<br>5′-TGCTTTTGTAGGCTTCAGTGGG-3′	180	60
GIP	NM_008119.2	5′-GTGGCTTTGAAGACCTGCTC-3′<br>5′-TTGTTGTCGGATCTTGTCCA-3′	186	56
Osteoclast-derived coupling factors				
LIF	NM_008501.2	5′-AGTAGCCGACTGCAGCTCTC-3′<br>5′-GACAGCTGTGCTGGATCAAA-3′	235	59
Cystatin C	NM_009976.4	5′-AAAGGCACACACTCCCTGAC-3′<br>5′-CCTGCAGCAGCTCCTTTACT-3′	188	60
Cell cycle- and apoptosis-related genes				
Cyclin D1	NM_001379248.1	5′-CAGAAGTGCGAAGAGGAGGTC-3′<br>5′-TCATCTTAGAGGCCACGAACAT-3′	130	56
Cdkn1a	NM_001111099.2	5′-GGTGGGCTTATCTGGGATGG-3′<br>5′-ATGTTTGGGGCTGGAGTCAG-3′	207	58
Cdkn2a	NM_009877.2	5′-ACGTTCACGTAGCAGCTCTT-3′<br>5′-TCTTGATGTCCCCGCTCTTG-3′	208	58
Bcl-2	NM_009741.5	5′-ATGCCTTTGTGGAACTATATGGC-3′<br>5′-GGTATGCACCCAGAGTGATGC-3′	120	56
Housekeeping genes				
β-actin	NM_007393.3	5′-CCAGGTCATCACTATTGGCA-3′<br>5′-ACCACCAGACAGCACTGTGTT-3′	172	54
18S rRNA	NR_003278.3	5′-GTAACCCGTTGAACCCCATT-3′<br>5′-CCATCCAATCGGTAGTAGCG-3′	151	57

Runx2, *runt*-related transcription factor-2; ALP, alkaline phosphatase; RANKL, receptor activator of nuclear factor-κB ligand; M-CSF, macrophage colony-stimulating factor; RANK, receptor for receptor activator of nuclear factor-κB ligand; Calr, Calcitonin receptor; TRAP, tartrate-resistant acid phosphatase; Atp6V0d2, ATPase H^+ Transporting V0 Subunit D2; NFATc1, nuclear factor of activated T-cells c1; DPP4, dipeptidyl peptidase 4; NPY, Neuropeptide Y; VIP, vasoactive intestinal peptide; Sub P, Substance P; IGF-1, insulin-like growth factor-1; GIP, glucose-dependent insulinotropic polypeptide; LIF, leukemia inhibitory factor; Cdkn1a, cyclin-dependent kinase inhibitor 1A; Cdkn2a, cyclin-dependent kinase inhibitor 2A.
